# Supplementary material for: Lysyl-tRNA synthetase, a target for urgently needed M. tuberculosis drugs
Source: Nat Commun. 2022 Oct 11;13:5992. doi: 10.1038/s41467-022-33736-5 (PMC9552147; doi:10.1038/s41467-022-33736-5)
Supplement: Supplementary file 1 — Supplementary Information [file 41467_2022_33736_MOESM1_ESM.pdf]

Supplementary Information for

Lysyl-tRNA synthetase, a target for urgently needed *M. tuberculosis* drugs

Simon R. Green[1], Susan H. Davis[1], Sebastian Damerow[1], Curtis A. Engelhart[2], Michael Mathieson[1], Beatriz Baragaña[1], David A. Robinson[1], Jevgenia Tamjar[1], Alice Dawson[1], Fabio K. Tamaki[1], Kirsteen I. Buchanan[1], John Post[1], Karen Dowers[1], Sharon M. Shepherd[1], Chimed Jansen[1], Fabio Zuccotto[1], Ian H. Gilbert[1], Ola Epemolu[1], Jennifer Riley[1], Laste Stojanovski[1], Maria Osuna-Cabello[1], Esther Pérez-Herrán[3], María José Rebollo[3], Laura Guijarro López[3], Patricia Casado Castro[3], Isabel Camino[3], Heather C. Kim[2], James M. Bean[4], Navid Nahiyaan[2], Kyu Y. Rhee[2], Qinglan Wang[5], Vee Y. Tan[5], Helena I. M. Boshoff[5], Paul J. Converse[6], Si-Yang Li[6], Yong S. Chang[6], Nader Fotouhi[7], Anna M. Upton[7], Eric L. Nuermberger[6], Dirk Schnappinger[2], Kevin D. Read[1], Lourdes Encinas[3], Robert H Bates[3], Paul G Wyatt[1] and Laura A.T. Cleghorn[1]*

[1]Drug Discovery Unit, Wellcome Centre for Anti-Infectives Research, Division of Biological Chemistry and Drug Discovery, College of Life Sciences, University of Dundee, Dundee, DD1 5EH, UK
[2]Dept. of Microbiology and Immunology, Weill Cornell Medical College, New York, New York, USA
[3]Global Health Medicines R&D, GlaxoSmithKline, Severo Ochoa 2, Tres Cantos, 28760, Madrid, Spain
[4]Sloan Kettering Institute, Memorial Sloan Kettering Cancer Center, 1275 York Avenue, New York, New York, USA
[5]Tuberculosis Research Section, Laboratory of Clinical Immunology and Microbiology, NIAID, NIH, 9000 Rockville Pike, Bethesda, Maryland, USA
[6]Center for Tuberculosis Research, Department of Medicine, Johns Hopkins University, School of Medicine, Baltimore, Maryland, USA
[7]Global Alliance for TB Drug Development, New York, New York, USA
*corresponding author (l.a.t.cleghorn@dundee.ac.uk)

Contains:

Supplementary Methods
Supplementary Figures
Supplementary Tables
Supplementary References

Chemical Synthesis

General Synthetic Routes

Flash chromatography was performed using a Combiflash Companion Rf (commercially available from Teledyne ISCO) and prepacked silica gel columns purchased from Teledyne ISCO. Mass-directed preparative HPLC separations were performed using a Waters HPLC (2545 binary gradient pumps, 515 HPLC make up pump, 2767 sample manager) connected to a Waters 2998 photodiode array and a Waters 3100 mass detector. Preparative HPLC separations were performed with a Gilson HPLC (321 pumps, 819 injection module, 215 liquid handler/injector) connected to a Gilson 155 UV/vis detector. On both instruments, HPLC chromatographic separations were conducted using Waters XBridge C18 columns, 19 x 100 mm, 5 um particle size; using 0.1% ammonia in water (solvent A) and acetonitrile (solvent B) or 0.1% formic acid in water (solvent A) and acetonitrile (solvent B) as mobile phase. ^{1}H-NMR spectra were recorded on a Bruker Avance DPX 500 spectrometer (^{1}H at 500.1 MHz, ^{13}C at 125 MHz ^{19}F at 470.5 MHz), Bruker Avance DPX 400 (1H at 400 MHz) or a Bruker Avance DPX 400 (1H at 300 MHz). Chemical shifts (δ) are expressed in p.p.m recorded using the residual solvent as the internal reference in all cases. Signal splitting patterns are described as singlet (s), doublet (d), triplet (t), quartet (q), multiplet (m), broad (br), or a combination thereof. Coupling constants (J) are quoted to the nearest 0.1 Hz. Low resolution electrospray (ES) mass spectra were recorded on an Advion Compact Mass Spectrometer (CMS; model ExpressIon CMS) connected to Dionex Ultimate 3000 UPLC system with diode array detector. HPLC chromatographic separations were conducted using a Waters XBridge C18 column, 2.1 x 50mm, 3.5 µm particle size or Waters XSelect 2.1 x 30mm, 2.5 µm particle size. The compounds were eluted with a gradient of 5 to 95% acetonitrile/water +0.1% Ammonia or +0.1% formic acid. High resolution mass spectroscopy (HRMS) was performed using a Bruker MicroTof mass spectrometer or Thermo Exploris 120 Orbitrap. Unless otherwise stated herein reactions have not been optimised. Solvents/reagents were purchased from commercial suppliers and used without further purification. Dry solvents were purchased in sure sealed bottles stored over molecular sieves.

Synthesis of 1

Compound **1** was commercially sourced from Princeton BioMolecular Research.

Synthesis of 2 (see Supplementary Figure 4)

4,6-Difluoro-1-hydroxyfuro[3,4-c]pyridin-3(1*H*)-one (11). *n*BuLi (2.5 M, 166 mL) was added dropwise to a solution of tetramethylpiperidine (61.7 g, 437 mmol) in THF (374 mL) under N_2 at -50°C. The mixture stirred at -50°C for 0.5 h then compound **10** (22 g, 138 mmol) was added as a solution in THF (110 mL). The mixture stirred at -50°C for an additional 0.5 h then DMF (53 mL, 691 mmol) was added and the reaction warmed to rt for 3 h and then poured onto ice (1 L) before 4N HCl (80 mL) and EtOAc were added. The organics were separated and washed with brine, dried over Na_2SO_4, filtered and concentrated *in vacuo*. The reaction was purified by reverse phase column chromatography (5-95% MeCN in water, 0.1% ammonium bicarbonate buffer) to afford **11** (16 g, 56% yield) as a yellow oil. ^{1}H-NMR (400 MHz, $CDCl_3$) *δ* 7.13 p.p.m. (d, *J* = 2.4 Hz, 1H), 6.68 (br s, 1H). MS (ES+) *m/z* 188.0 $[M + H]^+$

2-Cyclohexyl-4,6-difluoro-1,2-dihydro-3*H*-pyrrolo[3,4-*c*]pyridin-3-one (12). AcOH (15 mL, 262 mmol) and $NaBH(OAc)_3$ (19.82 g, 93.5 mmol) were added to a solution of **11** (7.42 g, 74.8 mmol) and cyclohexylamine (8.56 mL, 74.8 mmol) in DCM (70 mL) and the reaction stirred at

40°C for 16 h. EtOAc and water were added before the organics were separated, washed with water and brine, dried over Na_2SO_4 and filtered before concentrating *in vacuo*. The residue was purified by column chromatography (0.05 – 50% EtOAc in Petroleum ether) to afford **12** (2.1 g, 21% yield) as a yellow solid. ^{1}H-NMR (300 MHz, CD_3OD) δ 7.19 p.p.m. (d, J = 0.8 Hz, 1H), 4.61 (s, 2H), 4.00 - 4.15 (m, 1H), 1.94 - 1.69 (m, 5H), 1.66 - 1.40 (m, 4H), 1.36 - 1.17 (m, 1H). MS (ES+) *m/z* 253.1 $[M + H]^+$

2-Cyclohexyl-6-((2,4-dimethoxybenzyl)amino)-4-fluoro-1,2-dihydro-3*H*-pyrrolo[3,4-*c*]pyridin-3-one (13). DIPEA (2.90 mL, 16.7 mmol) was added to a solution of **12** (2.1 g, 8.3 mmol) and (2,4-dimethoxyphenyl)methanamine (1.39 g, 8.3 mmol) in DMF (20 mL), the reaction stirred at rt for 12 h. EtOAc and water were added, the organics separated, washed with water and brine, dried over Na_2SO_4 and filtered before concentrating *in vacuo* The residue was purified by column chromatography (0.05 – 75% EtOAc in Petroleum ether) to afford **13** (200 mg, 6% yield) as a white solid. ^{1}H-NMR (400 MHz, DMSO-d_6) δ 7.73 p.p.m. (t, J = 5.6 Hz, 1H), 7.10 (d, J = 8.4 Hz, 1H), 6.56 (d, J = 2.4 Hz, 1H), 6.46 (dd, J = 2.4, 8.4 Hz, 2H), 4.33 (s, 4H), 3.80 (s, 3H), 3.73 (s, 3H), 1.81 - 1.58 (m, 5H), 1.50 - 1.13 (m, 5H). MS (ES+) *m/z* 400.3 $[M + H]^+$

6-Amino-2-cyclohexyl-4-fluoro-1,2-dihydro-3*H*-pyrrolo[3,4-*c*]pyridin-3-one (2). A solution of **13** (200 mg, 5 mmol) in DCM (10mL) and TFA (2 mL) was stirred at rt for 3 h. The reaction was concentrated *in vacuo* and the residue was adjusted to pH 7 with sat. aq. $NaHCO_3$ and was purified by reverse phase column chromatography (26 - 56% MeCN in water, 0.05% ammonia hydroxide) to afford **2** (50 mg, 38% yield) as a white solid. ^{1}H-NMR (400 MHz DMSO-d_6) δ 6.90 p.p.m. (s, 2H), 6.37 (d, J = 2.5 Hz, 1H), 4.32 (s, 2H), 3.92 - 3.76 (m, 1H), 1.81 - 1.57 (m, 5H), 1.49 - 1.26 (m, 4H), 1.16 - 1.03 (m, 1H). ^{19}F-NMR (470 MHz, DMSO-d_6) δ -72.33. ^{13}C-NMR (126 MHz, DMSO-d_6) δ 163.0 (d, J_{FC} = 6.3 Hz), 160.8, 160.6, 158.4, 156.9 (d, J_{FC} = 5.4 Hz), 156.5, 98.4 (d, J_{FC} = 4.7 Hz), 49.7, 45.3, 30.4, 25.1. HRMS (*m/z*): $[M]^+$ calcd for $C_{13}H_{17}N_3OF$, 250.1355 found 250.1350

Synthesis of 18 and 19 (see Supplementary Figure 5)

Ethyl 4,6-dichloro-2-(methylthio)pyrimidine-5-carboxylate (15). Prepared as previously reported[1], using **14** (50 g, 256 mmol) to afford **15** (40.3 g, 59% yield) as a white solid. ^{1}H-NMR (500 MHz, $CDCl_3$) δ 4.48 p.p.m. (q, J = 7.1 Hz, 2H), 2.61 (s, 3H), 1.43 (t, J = 7.1 Hz, 3H). MS (ES+) *m/z* 267.1 $[M + H]^+$

Ethyl 4-methoxy-2-(methylthio)-6-vinylpyrimidine-5-carboxylate (16). *Step 1.* NaOMe (25 wt. % in MeOH, 11 mL, 47.6 mmol) was added dropwise to a solution of **15** (12.1 g, 45.4 mmol) in THF (130 mL) and cooled to 0°C. The reaction was stirred at 0°C for 30 min, then diluted with water and EtOAc. The combined organics were washed with brine, dried over $MgSO_4$ and concentrated *in vacuo* to afford ethyl 4-chloro-6-methoxy-2-(methylthio)pyrimidine-5-carboxylate that was taken onto the next step without further purification. MS (ES+) *m/z* 263.1 $[M + H]^+$

Step 2. DIPEA (14 mL, 81 mmol) was added to a solution of ethyl 4-chloro-6-methoxy-2-(methylthio)pyrimidine-5-carboxylate (11.83 g, 45.0 mmol) in anhydrous EtOH (130 mL) before the solution was degassed and flushed with N_2 three times. Potassium trifluoro(vinyl)borate (9 g, 67.6 mmol) and $Pd(dppf)_2Cl_2$.DCM (1.84 g, 2.3 mmol) were added and the reaction was degassed and flushed with N_2 again and then heated at 80°C for 16 h. The reaction mixture was concentrated *in vacuo* and the crude material was diluted with sat. aq. $NaHCO_3$ solution and EtOAc. The combined organics were washed with water, dried over $MgSO_4$ and concentrated *in vacuo*. The crude compound was purified by column

chromatography (0 – 10% EtOAc in heptane) to afford **16** (8.22 g, 67% yield over the two steps), as a colourless oil that crystallised in the fridge. ^{1}H-NMR (400 MHz, $CDCl_3$) δ 6.79 p.p.m. (dd, J = 16.8, 10.3 Hz, 1H), 6.66 (dd, J = 16.8, 2.1 Hz, 1H), 5.65 (dd, J= 10.3, 2.1 Hz, 1H), 4.38 (q, J = 7.1 Hz, 2H), 4.0 (s, 3H), 2.58 (s, 3H), 1.37 (t, J = 7.1 Hz, 3H). MS (ES+) m/z 255.2 $[M + H]^+$

Ethyl 4-formyl-6-methoxy-2-(methylthio)pyrimidine-5-carboxylate (18). $K_2O_4Os.2H_2O$ (102 mg, 0.3 mmol), $NaIO_4$ (6.7 g, 31.8 mmol) and 2,6-lutidine (1.85 mL, 15.9 mmol) were added **to a solution of 16** (2 g, 7.9 mmol) in THF (60 mL) and water (6 mL) and stirred for 16 h at rt. The reaction was diluted with EtOAc and water and the combined organics were washed with brine, dried over $MgSO_4$ and concentrated *in vacuo*. The crude material was purified by column chromatography (0 – 20% EtOAc in heptane). The impure fractions were purified by SCX. Compound **18** (1.09 g, 50% yield) was obtained as a yellow oil. ^{1}H-NMR (500 MHz, $CDCl_3$) δ 9.89 p.p.m. (s, 1H), 4.42 (q, J = 7.1 Hz, 2H), 4.07 (s, 3H), 2.62 (s, 3H), 1.37 (t, J = 7.1 Hz, 3H). MS (ES+) m/z 257.2 $[M + H]^+$

Ethyl 4-ethoxy-2-(methylthio)-6-vinylpyrimidine-5-carboxylate (17). *Step 1.* Sodium ethanolate (21% in EtOH, 49 mL, 130 mmol) was added dropwise over 55 min, to a cooled solution (2°C) of **15** (34.8 g, 130 mmol) in THF (270 mL), under N_2 and stirred for 45 min. The reaction was diluted with TBME and water. The combined organics were washed with brine, dried over Na_2SO_4 and concentrated *in vacuo* to afford ethyl 4-chloro-6-ethoxy-2-(methylthio)pyrimidine-5-carboxylate. Taken onto the next step without further purification. MS (ES+) m/z 277.2 $[M + H]^+$.

Step 2. TEA (21.4 mL, 154 mmol) was added **to a solution of** ethyl 4-chloro-6-ethoxy-2-(methylthio)pyrimidine-5-carboxylate (37.4 g, 135 mmol) and potassium vinyltrifluoroborate (23.4 g, 175 mmol) in EtOH (300 mL). N_2 was bubbled through the mixture for 10 min before addition of $Pd(dppf)_2Cl_2$.DCM (2.2 g, 2.70 mmol). The resulting mixture was refluxed under N_2 for 3 h before further addition of potassium vinyltrifluoroborate (12 g, 89.6 mmol), TEA (11 mL, 78.9 mmol) and $Pd(dppf)_2Cl_2$.DCM (1.1 g, 1.35 mmol) and heating continued for 16 h. The reaction was cooled and concentrated *in vacuo*. The residue was treated with 2N HCl, TBME was added and the combined organics were washed with sat. aq. $NaHCO_3$ followed by brine, dried over $MgSO_4$ and concentrated *in vacuo*. The residue was dissolved in DCM and filtered through a pad of silica to afford **17** (18.5 g, 51% yield over the two steps) as a pale brown oil. ^{1}H-NMR (400 MHz, $CDCl_3$) δ 6.80 p.p.m. (dd, J = 16.8, 10.5 Hz, 1H), 6.64 (dd, J = 16.8, 2.0 Hz, 1H), 5.64 (dd, J = 10.5, 2.0 Hz, 1H), 4.46 (q, J = 7.1 Hz, 2H), 4.38 (q, J = 7.1 Hz, 2H), 2.56 (s, 3H), 1.39 – 1.35 (m, 6H). MS (ES+) m/z 269.4 $[M + H]^+$

Ethyl 4-ethoxy-6-formyl-2-(methylthio)pyrimidine-5-carboxylate (19). Water (93 mL), $NaIO_4$ (35.7 g, 166.9 mmol) and OsO_4 (7.6 ml, 1.25 mmol, 4% aq. solution) were added to a solution of **17** (11.2 g, 41.74 mmol) and 2,6-lutidine (9.7 ml, 83.5 mmol) in 1,4-dioxane (742 ml). The reaction was stirred at rt for 2 h, diluted with water and DCM, passed through a hydrophobic frit and concentrated *in vacuo*. The crude compound was purified by column chromatography (0 – 15% EtOAc in heptane) to afford **19** (4.1g, 35% yield) as a brown oil. ^{1}H-NMR (400 MHz, DMSO-d_6) δ 9.80 p.p.m. (s, 1H), 4.51 (q, J = 7.1 Hz, 2H), 4.31 (q, J = 7.1 Hz, 2H), 2.59 (s, 3H), 1.32 (t, J = 7.1 Hz, 3H), 1.27 (t, J = 7.1 Hz, 3H). MS (ES+) m/z 71.2 $[M + H]^+$

Synthesis of 3 (see Supplementary Figure 6)

2-Amino-6-cyclohexyl-4-ethoxy-6,7-dihydro-5*H*-pyrrolo[3,4-*d*]pyrimidin-5-one (3). *Step 1*. Compound **19** (106 mg, 0.39 mmol) and cyclohexylamine (0.07 mL, 0.59 mmol) were combined in THF (3 mL) and stirred at rt for 5 min. STAB (249 mg, 1.18 mmol) was then added

and the reaction heated at 35 °C for 16 h. The reaction diluted with water and EtOAc and the organics were washed with brine, dried over Na_2SO_4, and concentrated *in vacuo*. The residue was purified by column chromatography (0 - 50% EtOAc in heptane) to afford 6-cyclohexyl-4-ethoxy-2-(methylthio)-6,7-dihydro-5*H*-pyrrolo[3,4-*d*]pyrimidin-5-one (77mg, 0.25 mmol) that was taken onto the next step without further purification. MS (ES+) *m/z* 308.4 $[M + H]^+$

Step 2. 6-cyclohexyl-4-ethoxy-2-(methylthio)-6,7-dihydro-5*H*-pyrrolo[3,4-*d*]pyrimidin-5-one (77mg, 0.25 mmol) and oxone (308 mg, 0. 50 mmol) were taken up in MeCN (2 mL) and water (1mL) then stirred at rt for 0.5 h. The reaction was diluted with EtOAc and the organics were washed with brine, dried over $MgSO_4$, and concentrated *in vacuo*. NH_3 in (0.5 M in 1,4-dioxane, 2 mL) was added, the reaction was sealed and heated at 105°C for 16 h. The volatiles were removed *in vacuo* and the residue purified by reverse phase column chromatography (5-95% MeCN in water, 0.1% NH_4OH) to afford **3** (41mg, 36% yield over the two steps) as a white solid. ^{1}H-NMR (500 MHz, DMSO-d_6) δ 7.15 p.p.m. (s, 2H), 4.39 (q, J = 7.1 Hz, 2H), 4.13 (s, 2H), 3.9 – 3.83 (m, 1H), 1.78 – 1.75 (m, 2H), 1.66 – 1.61 (m, 3H), 1.49 – 1.41 (m, 2H), 1.37 – 1.29 (m, 5H), 1.16 – 1.06 (m, 1H). ^{13}C-NMR (126 MHz, DMSO-d_6) δ 175.9, 164.9, 164.7, 163.9, 100.0, 61.3, 49.2, 46.5, 30.4, 25.2, 25.0, 14.3. HRMS (*m/z*): $[M]^+$ calcd for $C_{14}H_{21}N_4O_2$, 277.1664 found 277.1659.

Synthesis of 4 (see Supplementary Figure 6)

2-Amino-6-((1*R*,2*S*)-2-hydroxycyclohexyl)-4-methoxy-6,7-dihydro-5*H*-pyrrolo[3,4-*d*]pyrimidin-5-one (4). *Step 1.* Compound **18** (150 mg, 0.56 mmol), (1*S*,2*R*)-2-aminocyclohexanol hydrochloride (149 mg, 0.98 mmol) and DIPEA (0.16 mL, 0.58 mmol) were solubilised in DCM and stirred at rt for 1 h. STAB (372 mg, 1.76 mmol) was added and the reaction stirred at rt for 16 h, followed by 40°C for 8 h. Water was added and the organics were isolated by passing through a hydrophobic frit. Column chromatography (0 – 100% EtOAc in heptane) afforded 6-((1*R*,2*S*)-2-hydroxycyclohexyl)-4-methoxy-2-(methylthio)-6,7-dihydro-5*H*-pyrrolo[3,4-*d*]pyrimidin-5-one (153mg, 0.56 mmol) that was taken onto the next step without further purification. MS (ES+) *m/z* 310.3 $[M+H]^+$

Step 2. 6-((1*R*,2*S*)-2-hydroxycyclohexyl)-4-methoxy-2-(methylthio)-6,7-dihydro-5*H*-pyrrolo[3,4-*d*]pyrimidin-5-one (153mg, 0.56 mmol) and oxone (360 mg, 0. 59 mmol) were taken up in 1,4-dioxane (10 mL) and water (2.5 mL) and stirred at rt for 1 h. The reaction was diluted with water and DCM and the organics were isolated by passing through a hydrophobic frit and concentrated *in vacuo*. NH_3 in (0.5 M in 1,4-dioxane, 12 mL) was added to the residue, the reaction sealed and heated at 80°C for 4 h. The volatiles were removed *in vacuo* and purified by mass directed reverse phase column chromatography (5-95% MeCN in water, 0.1% NH_4OH) to afford **4** (29 mg, 17% yield, over the two steps) as a white solid. ^{1}H-NMR (400 MHz, $CDCl_3$) δ 5.29 p.p.m. (s, 2H), 4.53 (d, J = 18.9 Hz, 1H), 4.25 (br s, 1H), 4.21 (d, J = 18.9 Hz, 1H), 4.01 (s, 3H), 3.98 – 3.93 (m, 1H), 2.95 (br s, 1H), 2.11 (dq, J = 12.7, 3.8 Hz, 1H), 1.87 – 1.82 (m, 2H), 1.74 – 1.54 (m, 3H), 1.50 – 1.36 (m, 2H). ^{13}C-NMR (126 MHz, DMSO-d_6) δ 176.3, 165.3, 164.7, 164.2, 100.0, 67.8, 52.9, 52.4, 49.3, 32.6, 25.1, 24.1, 18.7. HRMS (*m/z*): $[M]^+$ calcd for $C_{13}H_{19}N_4O_3$, 279.1457 found 279.1449

Synthesis of 5 (see Supplementary Figure 6)

2-Amino-4-ethoxy-6-((1*R*,2*S*)-2-hydroxycyclohexyl)-6,7-dihydro-5*H*-pyrrolo[3,4-*d*]pyrimidin-5-one (5). *Step 1.* Compound **19** (1.2 g, 4.4 mmol), (1*S*,2*R*)-2-aminocyclohexanol hydrochloride (673 mg, 4.4 mmol) and DIPEA (0.77 mL, 4.4 mmol) were taken up in DCM (20 mL) and stirred at rt for 1 h. STAB (2.82 g, 13.3 mmol) was then added and the reaction stirred

at rt for 16 h, followed by 40°C for 8 h. The reaction was diluted with water and the organics isolated by passing through a hydrophobic frit and concentrating *in vacuo*. Column chromatography (0 – 100% EtOAc in heptane) afforded 4-Ethoxy-6-((1*R*,2*S*)-2-hydroxycyclohexyl)-2-(methylthio)-6,7-dihydro-5*H*-pyrrolo[3,4-*d*]pyrimidin-5-one (1.53 g, 4.6 mmol) that was taken onto the next step without purification. MS (ES+) *m/z* 324.4 $[M + H]^+$

Step 2. 4-ethoxy-6-((1*R*,2*S*)-2-hydroxycyclohexyl)-2-(methylthio)-6,7-dihydro-5*H*-pyrrolo[3,4-*d*]pyrimidin-5-one (1.53 g, 4.6 mmol) and oxone (2.79 g, 4.4 mmol) were taken up in 1,4-dioxane (10 mL) and water (2 mL) and stirred at rt for 1 h. The reaction was diluted with DCM and water and the organics isolated by passing through a hydrophobic frit and concentrated *in vacuo*. NH_3 (0.5M in 1,4-dioxane, 12 mL) was added to the residue, the reaction sealed and heated at 80°C for 4 h, volatiles removed *in vacuo.* Mass directed, reverse phase column chromatography (5-95% MeCN in water, 0.1% NH_4OH) afforded **5** (865 mg, 67% yield over the two steps) as a white solid. ^{1}H-NMR (400 MHz, $CDCl_3$) δ 5.19 p.p.m. (br s, 2H), 4.53 – 4.48 (m, 3H), 4.25 (br s, 1H), 4.2 (d, *J* = 18.7 Hz, 1H), 3.95 – 3.91 (m, 1H), 3.75 – 3.68 (m, 1H), 2.90 (d, *J* = 3.44 Hz, 1H), 2.12 (ddd, *J* = 25.2, 12.6, 3.5 Hz, 1H), 1.88 – 1.82 (m, 2H), 1.74 – 1.54 (m, 2H), 1.50 – 1.35 (m, 5H), 1.27 – 1.22 (m, 1H). ^{13}C-NMR (126 MHz, $CDCl_3$) δ 176.0, 166.2, 166.1, 164.4, 102.9, 69.7, 62.9, 55.9, 51.2, 33.1, 25.6, 24.5, 19.0, 14.4. HRMS (*m/z*): $[M]^+$ calcd for $C_{14}H_{21}N_4O_3$, 293.1608 found 293.1618.

Synthesis of 6 (see Supplementary Figure 6)

(*S*)-2-Amino-6-(2,2-difluorocyclohexyl)-4-methoxy-6,7-dihydro-5*H*-pyrrolo[3,4-*d*]pyrimidin-5-one (6). *Step 1.* Compound **18** (1.28 g, 5.0 mmol), (*S*)-2,2-difluorocyclohexan-1-amine hydrochloride (857 mg, 5.0 mmol) and DIPEA (1.3 mL, 7.5 mmol) were taken up in DCM (37 mL) and stirred at rt for 3 h. STAB (3.17 g, 14.9 mmol) was added and the reaction stirred at rt for 16 h. The reaction was diluted with water and the organics isolated by passing through a hydrophobic frit and concentrated *in vacuo*. Column chromatography (0 - 50% EtOAc in heptane) afforded (*S*)-6-(2,2-difluorocyclohexyl)-4-methoxy-2-(methylthio)-6,7-dihydro-5*H*-pyrrolo[3,4-*d*]pyrimidin-5-one (1.5 g, 4.55 mmol) that was taken onto the next step without further purification. MS (ES+) *m/z* 330.2 $[M+H]^+$

Step 2. (*S*)-6-(2,2-difluorocyclohexyl)-4-methoxy-2-(methylthio)-6,7-dihydro-5*H*-pyrrolo[3,4-d]pyrimidin-5-one (1.5 g, 4.55 mmol) and oxone (4.4 g, 7.27 mmol) were taken up in MeCN (27 mL) and water (18 mL) and stirred at rt for 3 h. The reaction was diluted with DCM and water and the organics isolated by passing through a hydrophobic frit and concentrated *in vacuo.* NH_3 (0.5M in 1,4-dioxane, 4 ml) was added to the residue and the reaction sealed and heated at 95°C for 16 h. The volatiles were removed *in vacuo* and mass directed, reverse phase column chromatography (5-95% MeCN in water, 0.1% NH_4OH) afforded **6** (648 mg, 43% yield over the two steps) as a white solid. ^{1}H-NMR (400 MHz, DMSO-d_6) δ 7.34 p.p.m. (s, 2H), 4.46 – 4.33 (m, 2H), 4.17 (d, *J* = 18.6 Hz, 1H), 3.90 (s, 3H), 2.11 – 2.04 (m, 1H), 1.99 – 1.70 (m, 5H), 1.55 – 1.37 (m, 2H). ^{19}F-NMR (470 MHz, DMSO-d_6) δ -98.13 (d, J_{FF} = 232.9 Hz), -111.04 (d, J_{FF} = 232.6 Hz). ^{13}C-NMR (126 MHz, DMSO-d_6) δ 176.0, 165.5, 165.1, 164.8, 123.9 (dd, J_{FC} = 248.1, 243.4 Hz), 99.0, 53.2, 51.5 (t, J_{FC} = 20.1 Hz), 48.4 (d, 4.9 Hz), 33.5 (t, J_{FC} = 23.3, 20.1 Hz), 27.5 (d, J_{FC} = 5.7 Hz), 23.4, 22.2 (d, J_{FC} = 9.6 Hz). HRMS (*m/z*): $[M]^+$ calcd for $C_{13}H_{17}N_4O_2F_2$, 299.1319 found 299.1314.

Synthesis of 7 (see Supplementary Figure 6)

2-Amino-6-cycloheptyl-4-ethoxy-6,7-dihydro-5*H*-pyrrolo[3,4-*d*]pyrimidin-5-one (7). *Step 1*. Compound **19** (225 mg, 0.8 mmol) and cycloheptanamine (0.16 mL, 1.3 mmol) were taken up

in THF (10 mL) and stirred at rt for 1 h. STAB (529 mg, 2.50 mmol) was added and stirred at rt for 4 h. The reaction was diluted with water and the organics isolated by passing through a hydrophobic frit and concentrated *in vacuo*. Column chromatography (0 - 50% EtOAc in heptane) afforded 6-cycloheptyl-4-ethoxy-2-(methylthio)-6,7-dihydro-5*H*-pyrrolo[3,4-*d*]pyrimidin-5-one (188 mg, 0.6 mmol) that was taken onto the next step without further purification. MS (ES+) *m/z* 322.1 $[M + H]^+$

Step 2. 6-Cycloheptyl-4-ethoxy-2-(methylthio)-6,7-dihydro-5*H*-pyrrolo[3,4-*d*]pyrimidin-5-one (188 mg, 0.6 mmol) was dissolved in MeCN (8 mL) and oxone (719 mg, 1.2 mmol) in water (4 mL) was added and the reaction stirred at rt for 3 h. The reaction was diluted with DCM and water and the organics isolated by passing through a hydrophobic frit and concentrated *in vacuo*. NH_3 (0.5 M in 1,4-dioxane, 4 mL, 8.8 mmol) was added, the reaction sealed and heated at 105°C, for 16 h. The volatiles were removed *in vacuo* and mass directed reverse phase column chromatography (5-95% MeCN in water, 0.1% NH_4OH) afforded **7** (96 mg, 39% yield over the two steps) as a white solid. ^{1}H-NMR (500 MHz, DMSO-d_6) δ 7.14 p.p.m. (br s, 2H), 4.39 (q, *J* = 7.0 Hz, 2H), 4.14 (s, 2H), 4.09 – 4.04 (m, 1H), 1.74 – 1.63 (m, 6H), 1.61 – 1.57 (m, 2H), 1.54 – 1.41 (m, 4H). ^{13}C-NMR (126 MHz, DMSO-d_6) δ 175.8, 164.9, 164.7, 163.6, 99.9, 61.2, 51.2, 46.5, 32.6, 27.3, 24.2, 14.3. HRMS (*m/z*): $[M]^+$ calcd for $C_{15}H_{23}N_4O_2$, 291.1821 found 291.1816.

Synthesis of 8 (see Supplementary Figures 7 - 9)

Di-*tert*-butyl (4-chloro-6-methoxypyrimidin-2-yl)carbamate (21). Prepared as per the referenced procedure[2], using compound **20** (50 g, 313 mmol) to afford **21** (60 g, 53% yield) as a colourless oil. ^{1}H-NMR (400 MHz, DMSO-d_6) δ 7.17 p.p.m. (s, 1H), 3.95 (s, 3H), 1.43 (s, 18H).

Methyl 2-((di-*tert*-butoxycarbonyl)amino)-4-chloro-6-methoxypyrimidine-5-carboxylate (22). To a solution of **21** (70 g, 194.5 mmol) in THF (1 L) was added LDA (2 M, 194.55 mL, 389 mmol) dropwise at -60°C under N_2. Methyl chloroformate (28.7 mL, 370 mmol) was added before the reaction stirred was at -40°C for 2 h. The mixture was quenched with sat. aq. NH_4Cl, EtOAc was added and the organics were separated, washed with brine, dried over Na_2SO_4, filtered and concentrated *in vacuo*. The reaction was repeated a further 3 times and all batches of crude products were combined and purified together by column chromatography (0 – 10% EtOAc in p**etroleum ether**) to afford **22** (290 g, 89% yield) as a yellow oil. ^{1}H-NMR (400 MHz, DMSO-d_6) δ 4.00 p.p.m. (s, 3H), 3.91 (s, 3H), 1.46 (s, 18H).

Methyl 2-((*tert*-butoxycarbonyl)amino)-4-chloro-6-methoxypyrimidine-5-carboxylate (23). A mixture of **22** (250 g, 598 mmol) and aq. K_3PO_4 (2 M, 598 mL) in 1,4-dioxane (2 L) were stirred at 90°C for 12 h. The reaction was poured onto ice-water (w/w = 1/1, 500 mL), stirred for 5 min, EtOAc added and the organics separated, washed with brine, dried over Na_2SO_4, filtered and concentrated *in vacuo*. The residue was purified by column chromatography (0 – 10% EtOAc in p**etroleum ether**) to afford **23** (130 g, 68% yield) as a white solid. ^{1}H-NMR (400 MHz, DMSO-d_6) δ 10.57 p.p.m. (s, 1H), 4.02 - 3.96 (m, 3H), 3.84 (s, 3H), 1.47 (s, 9H).

Methyl 2-((*tert*-butoxycarbonyl)amino)-4-methoxy-6-vinylpyrimidine-5-carboxylate (24). Compound **23** (30 g, 69 mmol) was dissolved in 1,4-dioxane (500 mL) before the addition of vinyl Bpin (24 mL, 141.5 mmol), Pd(dppf)Cl_2 (3 g, 4.10 mmol) and K_3PO_4 (30 g, 141 mmol) as a solution in water (100 mL) were added at 25°C under N_2 then the reaction was stirred at 80°C for 12 h. The reaction was then poured into ice-water (w/w = 1/1, 50 mL) and stirred for 5 min, EtOAc added and the organics separated, washed with brine, dried over Na_2SO_4, filtered and concentrated *in vacuo*. The residue was purified by column chromatography (0 – 10% EtOAc in p**etroleum ether**) to afford **24** (14.2 g, 57% yield, 86% purity) as a yellow solid. ^{1}H-

NMR (400 MHz, DMSO-d_6) δ 10.11 p.p.m. (s, 1H), 6.83 - 6.68 (m, 1H), 6.64 - 6.52 (m, 1H), 5.78 - 5.67 (m, 1H), 3.94 (s, 3H), 3.84 (s, 3H), 1.48 (s, 9H). MS (ES+) *m/z* 310.1 $[M+H]^+$

Methyl 2-((*tert*-butoxycarbonyl)amino)-4-formyl-6-methoxypyrimidine-5-carboxylate (25). 2,6-lutidine (7.5 mL, 64.66 mmol), $K_2OsO_4.2H_2O$ (417 mg, 1.13 mmol) and $NaIO_4$ (27.7 g, 129.3mmol) were added to a solution of **24** (10 g, 32.3 mmol) in THF (244.5 mL) and water (24.5 mL) and stirred for 16 h at rt, corn oil (10 mL) added and the reaction stirred for a further 1 h, concentrated *in vacuo* and EtOAc added, the organics separated, washed with brine, dried over $MgSO_4$, filtered and concentrated *in vacuo*. The residue was purified by column chromatography (0 – 50% EtOAc in heptane) to afford **25** (3.6 g, 36% yield) as a green viscous oil. ^{1}H-NMR (500 MHz, $CDCl_3$) δ 9.9 p.p.m. (s, 1H), 4.06 (s, 3H), 3.93 (s, 3H), 1.56 (s, 9H). *m/z* (ESI): 212.2$[M -BOC]^+$

2-Iodocyclohept-2-en-1-one (27). Prepared as per the referenced procedure[3], using compound **26** (100 mL, 907 mmol) to afford **27** (110 g, 51% yield) as a yellow oil. ^{1}H-NMR (400 MHz, $CDCl_3$) δ 7.64 - 7.60 p.p.m. (m, 1H), 2.74 – 2.71 (m, 2H), 2.44 – 2.42 (m, 2H), 1.85 – 1.78 (m, 4H).

(1*S*,7*S*)-8-((*R*)-1-Phenylethyl)-8-azabicyclo[5.1.0]octan-2-one (28). (*R*)-1-phenylethan-1-amine (90 mL, 699 mmol) and 1,10-phenanthroline (83.9 g, 466 mmol) were added to a solution of **27** (110 g, 466 mmol) in 2-methylbutan-2-ol (1 L). The mixture was heated to 95°C before the careful addition of Cs_2CO_3 (227 g, 699 mmol) under N_2 and the reaction stirred at 110°C for 16 h, cooled to rt and filtered through a pad of silica gel. The filter cake was washed with EtOAc (500 mL) and the filtrate was dried over Na_2SO_4 and concentrated *in vacuo*. The crude product was purified by column chromatography (0 – 5% EtOAc in petroleum ether) to afford **28** (65 g, 61% yield) as a yellow oil. ^{1}H-NMR (400 MHz, $CDCl_3$) δ 7.38 - 7.25 p.p.m. (m, 5H), 2.96 – 2.93 (m, 1H), 2.83 - 2.83 (m, 1H), 2.66 – 2.62 (m, 1H), 2.18 – 2.15 (m, 2H), 1.84 - 1.80 (m, 2H), 1.72 - 1.63 (m, 3H), 1.42 - 1.39 (m, 3H), 0.98 - 0.94 (m, 1H).

(1*S*,7*S*)-2,2-Difluoro-8-((*R*)-1-phenylethyl)-8-azabicyclo[5.1.0]octane (29). BAST (248 mL, 113 mmol) was added dropwise to a cooled to solution (0°C) of **28** (65 g, 283 mmol) in DCM (650 mL). The reaction was stirred at 40°C for 24 h, cooled to rt and poured onto ice water (1 L) slowly and washed with brine. The combined organics were dried over Na_2SO_4, filtered and concentrated *in vacuo* and purified by column chromatography (0 – 5% EtOAc in petroleum ether) to afford **29** (21 g, 29%, yield) as a yellow oil. ^{1}H-NMR (400 MHz, $CDCl_3$) δ 7.36 - 7.31 p.p.m. (m, 4H), 7.27 - 7.23 (m, 1H), 2.70 – 2.67 (m, 1H), 2.28 – 2.06 (m, 1H), 1.92 – 1.72 m, 4H), 1.62 – 1.59 (m, 4H), 1.40 – 1.38 (m, 3H), 1.17 - 1.14 (m, 1H).

(1*R*,2*S*)-3,3-Difluoro-2-(((*R*)-1-phenylethyl)amino)cycloheptan-1-ol (30). Aq. H_2SO_4, (9 M, 105 mL) was added to **29** (21 g, 83.5 mmol) at 20°C before the mixture was stirred at 100°C for 36 h. The mixture was cooled to rt and adjusted to pH 7 with aq. NaOH (8 M). The resulting mixture was filtered, washed with EtOAc and the combined organics were washed with brine, dried over Na_2SO_4 and concentrated *in vacuo*. The crude product was purified by column chromatography (4 – 20% EtOAc in petroleum ether) to afford **30** (25 g, 75% yield) as a yellow oil. ^{1}H-NMR (400 MHz, $CDCl_3$) δ 7.29 - 7.18 p.p.m. (m, 5H), 4.14 - 4.19 (m, 1H), 3.23 - 3.20 (m, 1H), 2.84 - 2.79 (m, 1H), 2.19 - 2.11 (m, 2H), 1.75 – 1.74 (m, 2H), 1.75 – 1.74 (m, 1H), 1.48 - 1.45 (m, 3H), 1.29 -1.28 (m, 3H).

(1*R*,2*S*)-2-Amino-3,3-difluorocycloheptan-1-ol 2,2,2-trifluoroacetate (31). $Pd(OH)_2$ (2.00 g, 14.2 mmol) and TFA (6.87 mL, 92.8 mmol) were added to a solution of **30** (25 g, 92.8 mmol) in MeOH (120 mL) under N_2. The suspension was degassed under vacuum and purged with

H_2 several times. The mixture was stirred under H_2 (15 psi) at 30°C for 2 h then filtered through celite and the filtrate was concentrated *in vacuo* to afford **31** (24 g, 93% yield) as a yellow oil. The compound was used without further purification. ^{1}H-NMR (400 MHz, CD_3OD) δ 3.73 - 3.70 p.p.m. (m, 1H), 3.59 - 3.51 (m, 1H), 2.32 - 2.03 (m, 3H), 1.81 - 1.67 (m, 5H).

***N*-((1*S*,7*R*)-2,2-Difluoro-7-hydroxycycloheptyl)acetamide (32).** TEA (18 mL, 129 mmol) and Ac_2O (9.66 mL, 103 mmol) were added dropwise to a cooled solution (0°C) of **31** (24 g, 86 mmol) in MeOH (250 mL). The mixture was stirred at 20°C for 16 h, concentrated *in vacuo* and purified by column chromatography (0 – 10% MeOH in DCM) to afford **32** (14g, 79% yield) as a white solid. ^{1}H-NMR (400 MHz, CD_3OD) δ 4.28 - 4.19 p.p.m. (m, 1H), 3.62 – 3.57 (m, 1H), 2.11 – 2.08 (m, 2H), 1.82 (s, 3H), 1.82 - 1.79 (m, 1H), 1.80 - 1.79 (m, 2H), 1.68 – 1.66 (m, 2H), 1.66 - 1.57 (m, 1H).

(3a*S*,8a*S*)-4,4-Difluoro-2-methyl-3a,5,6,7,8,8a-hexahydro-4*H*-cyclohepta[*d*]oxazole (33). $SOCl_2$ (9.80 mL, 135 mmol) was added to a cooled solution (0°C) of **32** (14 g, 67.5 mmol) in DCM (140 mL). The mixture was stirred at 20°C for 12 h and concentrated *in vacuo* to afford **33** (14.8 g, quantitative) as a white solid. The compound was used without further purification. ^{1}H-NMR (400 MHz, $CDCl_3$) δ 5.71 - 5.70 p.p.m. (m, 1H), 5.48 – 5.40 (m, 1H), 2.64 (s, 3H), 2.26 - 2.19 (m, 3H), 2.04 – 2.01 (m, 1H), 1.74 – 1.56 (m, 4H).

(1*S*,2*S*)-2-Amino-3,3-difluorocycloheptan-1-ol hydrochloride (34). Aq. HCl (12 M, 140 mL) was added to a solution of **33** (14 g, 74 mmol) in water (140 mL) and the reaction was stirred at 100°C for 12 h. The mixture was concentrated *in vacuo* to afford **34** (12.8 g, 86% yield) as a light yellow solid. The compound was used in subsequent steps without purification. ^{1}H-NMR (400 MHz, CD_3OD) δ 4.19 - 4.17 p.p.m. (m, 1H), 3.84 – 3.79 (m, 1H), 2.29 – 2.22 (m, 2H), 1.99 - 1.97 (m, 1H), 1.80 – 1.77 (m, 3H), 1.64 – 1.58 (m, 2H). MS (ES+) *m/z* 166.1 $[M + H]^+$

2-Amino-6-((1*S*,7*S*)-2,2-difluoro-7-hydroxycycloheptyl)-4-methoxy-6,7-dihydro-5*H*-pyrrolo[3,4-*d*]pyrimidin-5-one: DDD02049209 (8). *Step 1.* Compound **25** (7.3 g, 23.6 mmol) and (1S,2S)-2-amino-3,3-difluoro-cycloheptanol (3.9 g, 23.6 mmol) were taken up in DCM (225 mL), a spatula of $MgSO_4$ was added followed by the addition of STAB (14.9 g, 70.8 mmol), and the reaction was stirred at rt for 16 h. The reaction was diluted with DCM and water, the organic layer was collected using a hydrophobic frit and concentrated *in vacuo*. The residue was taken up in 200 mL of THF and refluxed for 16 h. The reaction was cooled to rt and concentrated *in vacuo*. The crude material was purified by column chromatography (0 – 70% EtOAc in heptane) to afford *tert*-butyl (6-((1*S*,7*S*)-2,2-difluoro-7-hydroxycycloheptyl)-4-methoxy-5-oxo-6,7-dihydro-5*H*-pyrrolo[3,4-*d*]pyrimidin-2-yl)carbamate that was taken onto the next step without further purification**.** MS (ES+) *m/z* 429.3 $[M+H]^+$

Step 2. TFA (6.4 mL) was added to a solution of *tert*-butyl (6-((1*S*,7*S*)-2,2-difluoro-7-hydroxycycloheptyl)-4-methoxy-5-oxo-6,7-dihydro-5*H*-pyrrolo[3,4-*d*]pyrimidin-2-yl)carbamate (7.18 g, 16.8 mmol) in DCM (250 mL) and stirred for 16 h at rt. Purification by SCX followed by crystallisation in EtOH afforded **8** (3.3 g, 59% yield over the two steps) as an off white solid. ^{1}H-NMR (400 MHz, DMSO-d_6) δ 7.34 p.p.m. (br s, 2H), 5.21 (d, J = 4 Hz, 2H), 4.61 – 5.52 (m, 1H), 4.34 (d, J = 18 Hz, 1H), 4.03 -4.01 (m, 2H), 3.91 (s, 3H), 2.21 – 2.07 (m, 2H), 1.90 – 1.88 (m, 1H), 1.77 – 1.71 (m, 1H), 1.65 – 1.59 (m, 3H), 1.49- 1.44 (m, 1H). ^{19}F-NMR (470 MHz, DMSO-d_6) δ – 82.71 (d, J_{FF} = 241.9 Hz), -93.7 (d, J_{FF} = 241.6 Hz). ^{13}C-NMR (126 MHz, $CDCl_3$) δ 175.4, 167.5, 166.6, 164.8, 102.5, 70.2 (d, J_{FC} = 10.3 Hz), 66.7 – 66.2 (m), 54.7 (d, J_{FC}

= 3.4 Hz), 54.2, 36.7 (t, J_{FC} = 24.4 Hz), 35.5, 24.2, 21.7, 21.6. HRMS (*m/z*): $[M]^+$ calcd for $C_{14}H_{19}N_4O_3F_2$, 329.1425 found 329.1420

Synthesis of 9 (see Supplementary Figure 10)

Ethyl 2-amino-4-(bromomethyl)pyrimidine-5-carboxylate (36). Bromine (0.28mL, 5.52 mmol) was added to a solution of **35** (1 g, 5.52 mmol) in AcOH (55 mL) and the reaction was heated at 60°C for 2 h, cooled to rt, and the volatiles were removed *in vacuo*. Sat. aq. $Na_2S_2O_3$ was added and the mixture filtered, washed with EtOAc and the combined organics were dried over Na_2SO_4 and concentrated *in vacuo.* The residue was purified by column chromatography (0 – 25% EtOAc in heptane) to afford **36** (450mg, 31% yield) as a white solid. ^{1}H-NMR (400 MHz, $CDCl_3$) δ 8.90 p.p.m. (s, 1H), 5.46 (brs, 2H), 4.77, (s, 2H), 4.38 (q, *J* = 7.13Hz, 2H), 1.40 (t, *J* = 7.13Hz, 3H). MS (ES+) *m/z* 260.1, 262.1 $[M+H]^+$

2-Amino-6-cyclobutyl-6,7-dihydro-5*H*-cyclopenta[*d*]pyrimidin-5-one (9). TEA (0.32 mL, 2.3 mmol) was added to a solution of cyclobutylamine (0.1 mL, 1.15 mmol) in NMP (1 mL) followed by portion wise addition of **36** (100 mg, 0.38 mmol), the reaction was stirred at rt for 15 min and then heated in a MW for 1 h at 130°C. Reverse phase column chromatography (5-95% MeCN in water, 0.1% NH_4OH) afforded **9** (28 mg, 34% yield) as a white solid. ^{1}H-NMR (400 MHz, DMSO-d_6) δ 8.47 p.p.m. (s, 1H), 7.36 (s, 2H), 4.73 – 4.64 (m, 1H), 4.40 (s, 2H), 2.34 – 2.23 (m, 2H), 211 – 2.05 (m, 2H), 1.71 – 1.62 (m, 2H). ^{13}C-NMR (126 MHz, $CDCl_3$) δ 173.3, 164.7, 164.5, 153.8, 113.5, 47.1, 45.1, 27.8, 14.5. HRMS (*m/z*): $[M]^+$ calcd for $C_{10}H_{13}N_4O$, 205.1089 found 205.1082

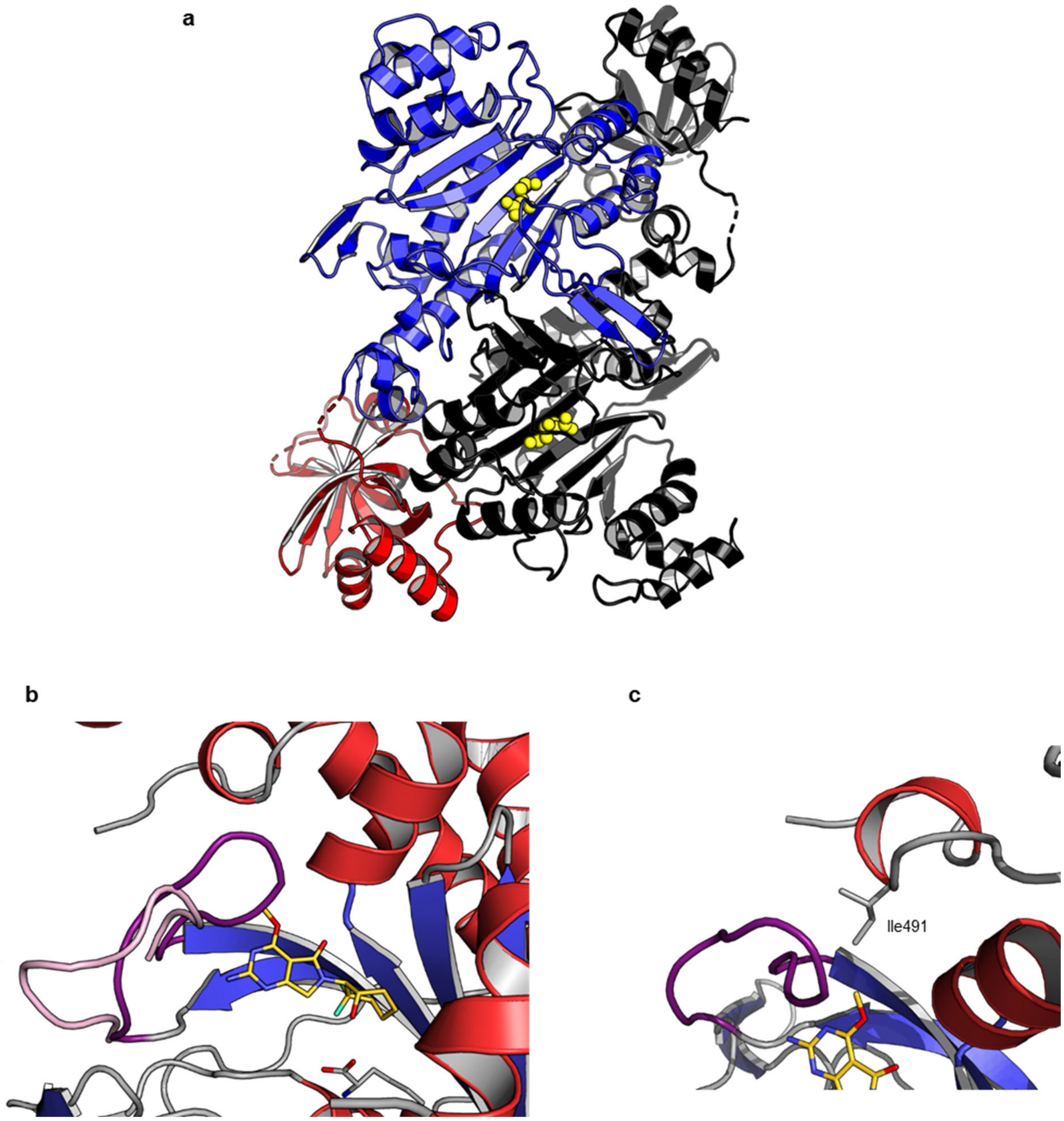

Supplementary Figure 1: Crystal structure of the LysRS dimer.
a. Crystal structure of the LysRS dimer. One monomer is shown with the N-terminal anti-codon binding domain in red, and the C-terminal catalytic domain in blue, with the bound lysine shown as yellow spheres. The crystallographically equivalent second monomer, which completes the physiological dimer, is shown in black. LysRS has high structural similarity to previously determined lysyl-tRNA synthetase structures, with high levels of secondary structure matching with human (KARS1 PDB 6ild; % of secondary structures elements matched [% sse] 87%), bacterial (*e.g. E. coli* LysRS; PDB 1e1t; % sse 90%), and apicomplexan KRS (*e.g. Plasmodium* LysRS; PDB 6agt; % sse 81%). **b**. Close up of the binding site of LysRS highlighting the two observed loop conformations. In the absence of a ligand in the adenosine pocket, residues 258-267 adopt an open conformation shown in light pink (PDB 7qh8). Upon ligand binding, the loop closes over the pocket as shown in dark purple (shown for PDB 7qi8). In KARS1, in the absence of ligand (PDB 6ild) the loop position is similar to the closed (dark purple) position of LysRS. **8** is shown in the binding pocket with the same colour scheme as Fig 2. **c.** Close up of the C-terminus of LysRS (showing PDB 7qi8). In the resistant strain, a serine is inserted before Ile491 (shown in sticks). The C-terminus of the protein caps the ligand/adenosine binding pocket.

a

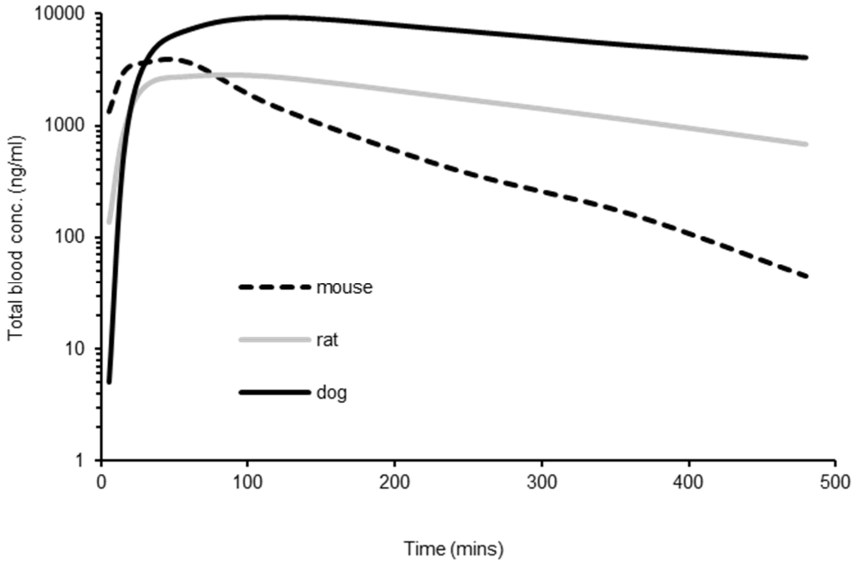

b

	Mouse		Rat		Dog	
	IV	PO	IV	PO	IV	PO
C_{max} (ng/mL)		3699		2800		9380
T_{max} (hr)		0.5		1		2
$T_{1/2}$ (hr)	1.4		2.0		7.4	
AUC0-480 (µg/mL.min)	201	499	345	821	984	3008
Clb (mL/min/kg)	15		10		2.3	
Vd_{ss} (L/kg)	1.0		1.4		0.9	
F (%)		74		83		105

Supplementary Figure 2: Cross species PK parameters for 8.

a. Comparative cross species pharmacokinetic profiles for **8**, dosed at 10 mg/kg PO in mouse, rat and dog. **b.** Comparative cross species pharmacokinetic parameters for **8**, dosed at 3mg/kg IV and 10 mg/kg PO in mouse, rat and dog. Source data are provided as a Source Data file.

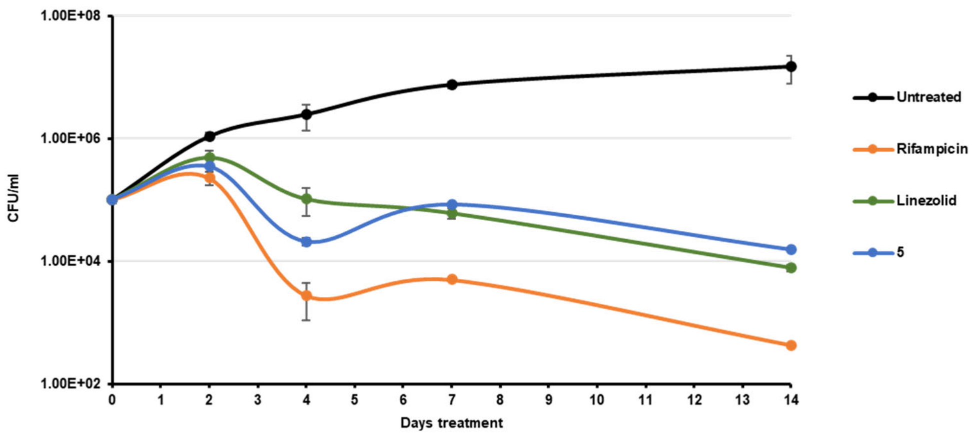

Supplementary Figure 3: Cidality of LysRS inhibitors.

Cidality, time kill kinetics for **5** as a representative of the LysRS inhibitor series in comparison with rifampicin and linezolid (all at 10x MIC). The experiment was performed twice with the graph showing representative data from one experiment run with duplicate samples and presented as mean values +/- standard deviation. Source data are provided as a Source Data file.

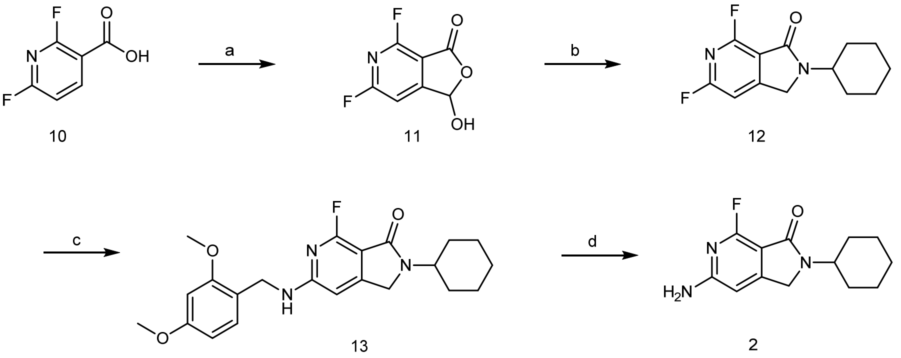

Supplementary Figure 4: Synthetic route to 2.
Reagents and conditions: (a) *n*BuLi, tetramethylpiperidine, THF, DMF, -50°C – rt, 4 h, 56%; (b) Cyclohexylamine, AcOH, $NaBH(OAc)_3$, DCM, 40°C, 16 h, 21%; (c) (2,4-dimethoxyphenyl)methanamine, DIPEA, rt, 12 h, 6%; (d) TFA, rt, 3 h, 38%.

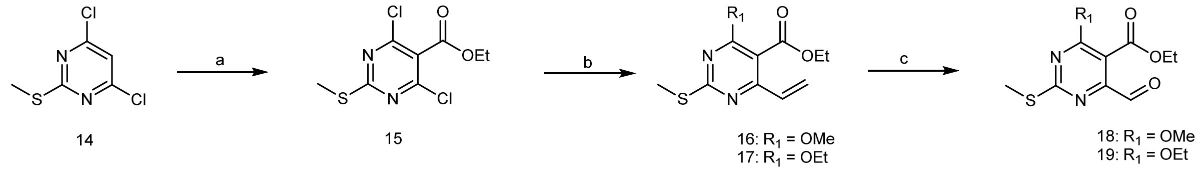

Supplementary Figure 5: Synthetic Route to 18 and 19.
Reagents and conditions; (a) prepared as in WO2011024871, 59%; (b) *Step 1.* NaOEt or NaOMe, THF, 0- 2°C, 30 – 45 min. *Step 2.* TEA, potassium vinyltrifluoroborate, $Pd(dppf)_2Cl_2$.DCM, EtOH, 80°C – reflux, 16 h, 51 – 70%; (c) OsO_4 or $K_2O_4Os.2H_2O$, $NaIO_4$, Water, 2,6-lutidine, rt, 2 – 16 h, 35 – 50%.

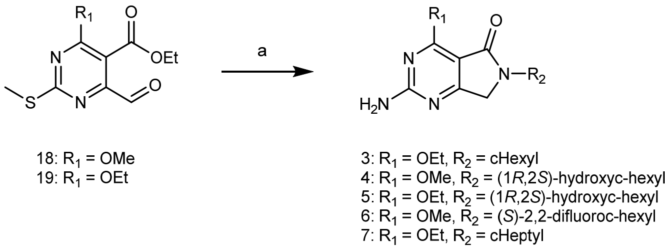

Supplementary Figure 6: Synthetic Route to 3 - 7.
(a) *Step 1.* i. R_2NH_2, TEA, $MgSO_4$, DCM, rt, 5 min – 3h ii. STAB, rt - 40°C, 4 – 16h *Step 2.* i. Oxone, MeCN or 1,4-dioxane, Water, rt, 0.5 – 3 h ii. NH_3 in (0.5 M in 1,4-dioxane), 80 - 105°C, 17 – 75% (over two steps).

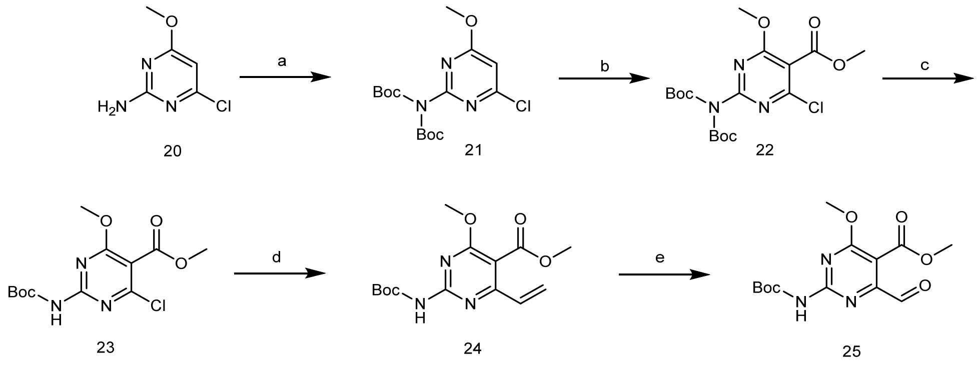

Supplementary Figure 7: Synthetic Route to 25.
Reagents and conditions; (a) prepared as in WO2008106692, 53%; (b) LDA, methyl chloroformate, THF, -60 - 40°C, 89%; (c) K_3PO_4, 1,4-dioxane, 90°C, 12 h, 68% (d) vinyl Bpin, Pd(dppf)Cl_2, K_3PO_4, 1,4-dioxane, water, 80°C, 12 h, 57% (e) $K_2OsO_4.2H_2O$, 2,6-lutidine, $NaIO_4$, rt, 16 h, 36%.

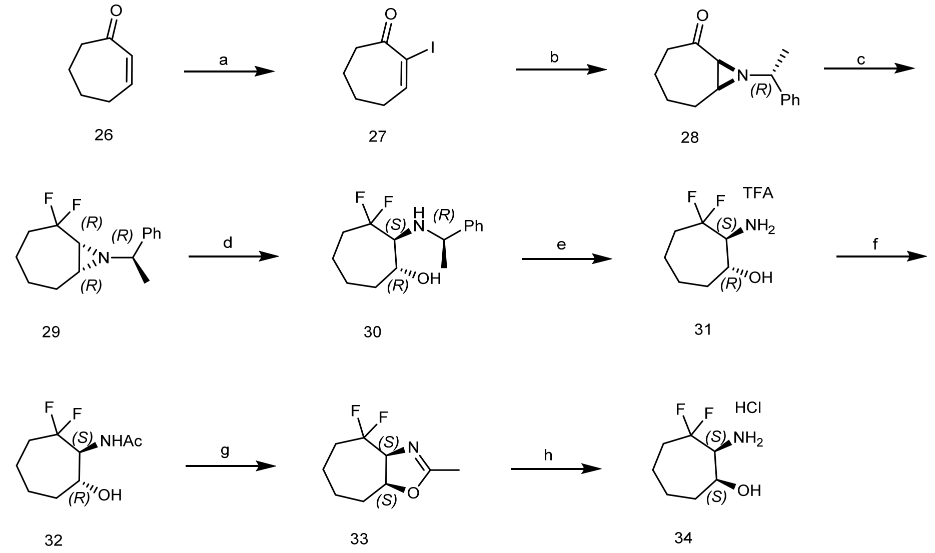

Supplementary Figure 8: Synthetic Route to 34.
Reagents and conditions; (a) prepared as in WO2011024871, 51%; (b) (*R*)-1-phenylethan-1-amine**,** 1,10-phenanthroline, Cs_2CO_3, 2-methylbutan-2-ol, 110 °C, 16 h, 61%; (c) BAST, DCM, 0 - 40°C, 24 h, 29%; (d) Aq. H_2SO_4, 20 - 100°C, 36 h, 75%; (e) $Pd(OH)_2$, TFA, H_2, MeOH, 30°C, 2 h, 93%; (f) Ac_2O, TEA, 20°C, 16 h, 79%; (g) $SOCl_2$, DCM, 0 - 20°C, 12 h, quantitative; (h) Aq. HCl, water, 100°C, 12 h, 86%.

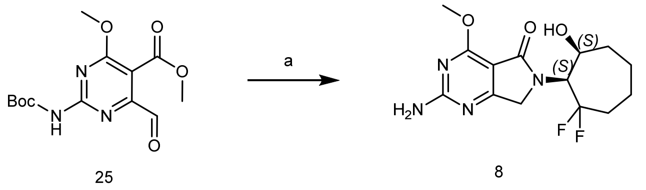

Supplementary Figure 9: Synthetic Route to 8.
Reagents and conditions; (a) *Step 1.* **34**, $MgSO_4$, STAB, DCM, rt, 16 h then THF, reflux, 16 h; *Step 2*. TFA, DCM, 16 h, rt, 59% (over the two steps).

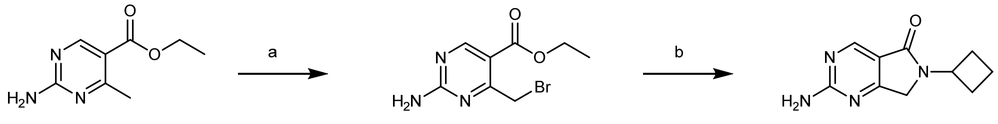

35 36 9

Supplementary Figure 10: Synthetic Route to 9.
Synthesis of **9**. Reagents and conditions; (a) Bromine, AcOH, 60°C, 2 h, 31%; (b) cyclobutylmine, TEA, NMP, MW, 130°C, 1 h, 34%.

Supplementary Figures 11 - 29 NMR Spectra for Compounds

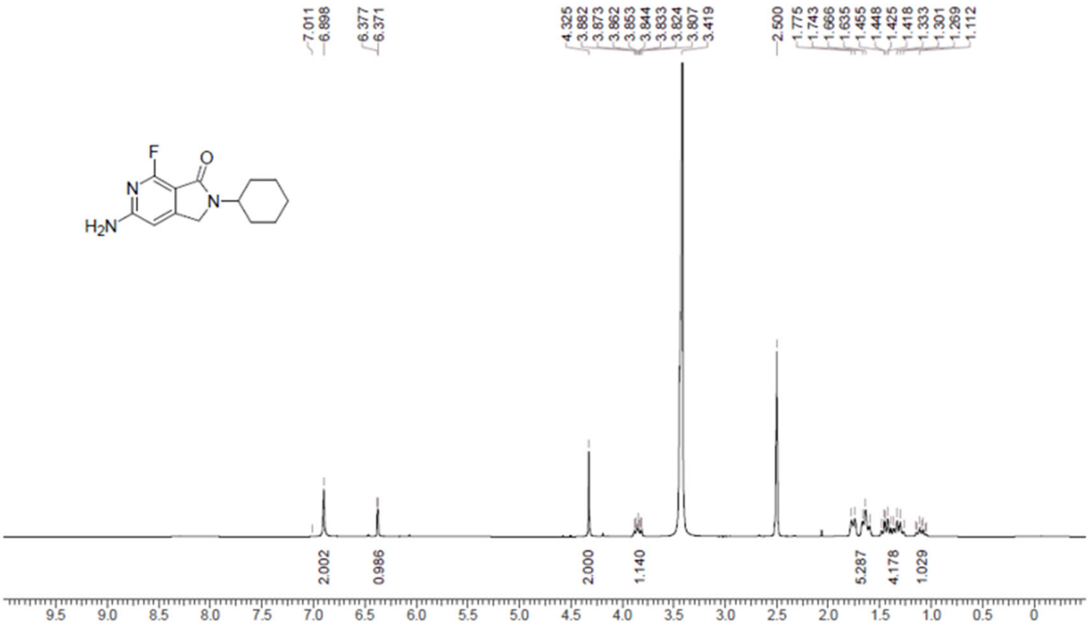

Supplementary Figure 11. ^{1}H NMR spectra of compound **2** (rt, in DMSO-d_6)

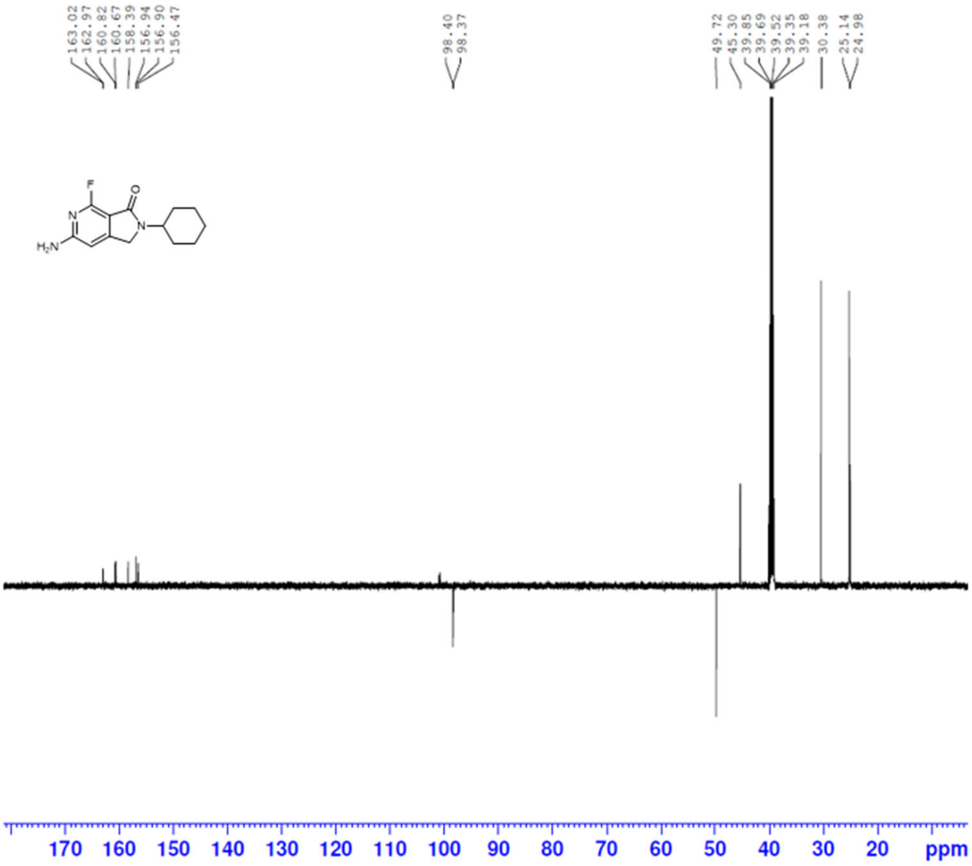

Supplementary Figure 12. DEPTQ NMR spectra of compound **2** (rt, in DMSO-d_6)

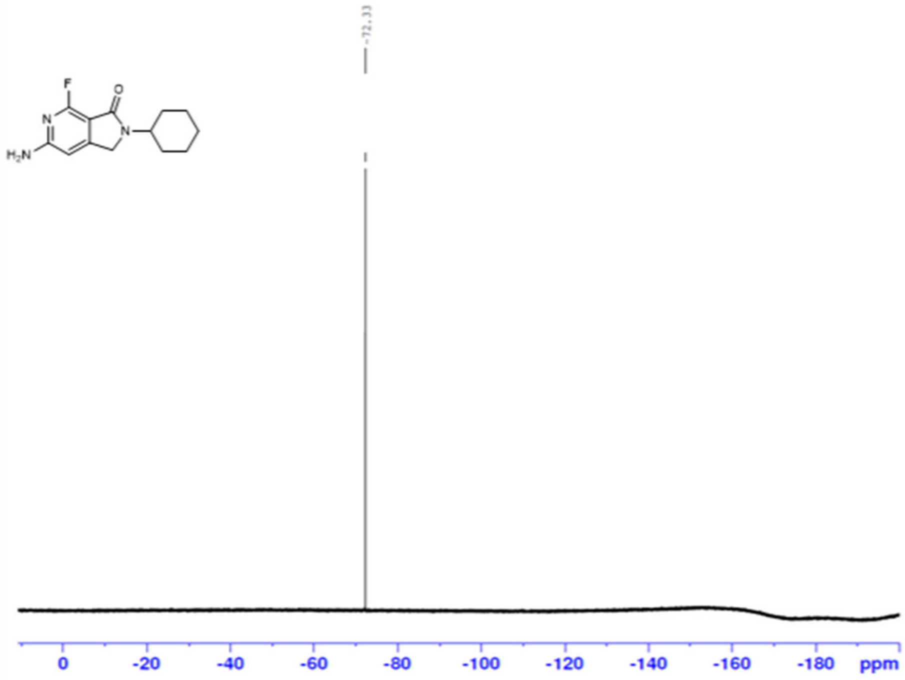

Supplementary Figure 13. ^{19}F NMR spectra of compound **2** (rt, in DMSO-d_6)

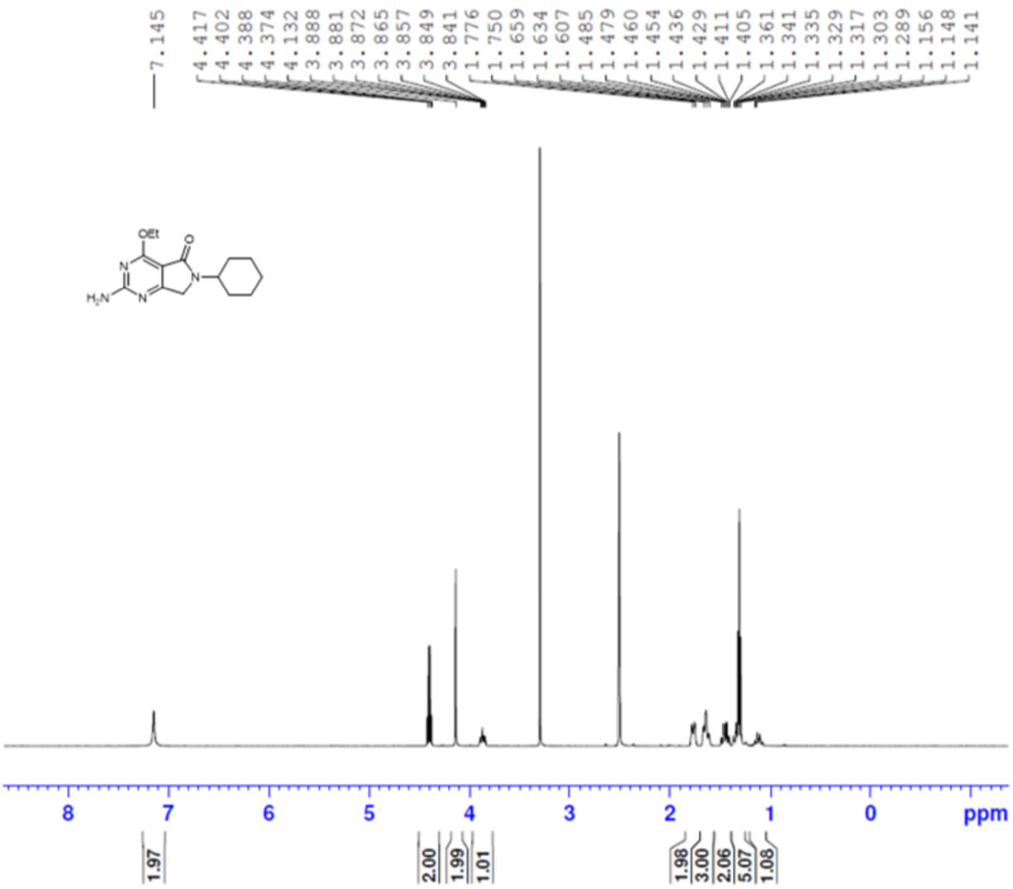

Supplementary Figure 14. ^{1}H NMR spectra of compound **3** (rt, in DMSO-d_6)

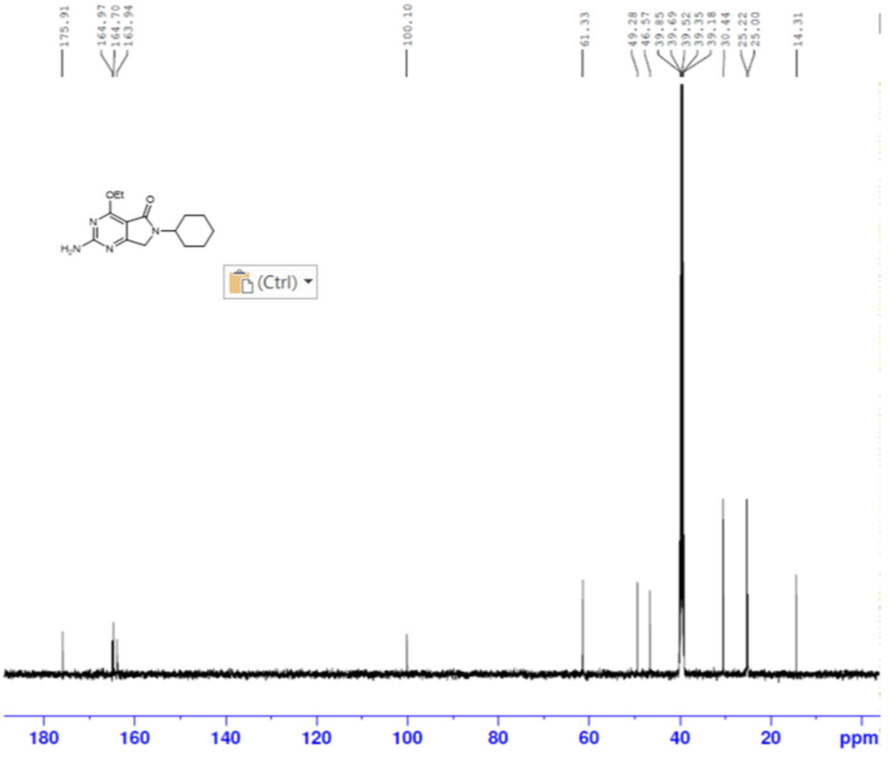

Supplementary Figure 15. ^{13}C NMR spectra of compound **3** (rt, in DMSO-d_6)

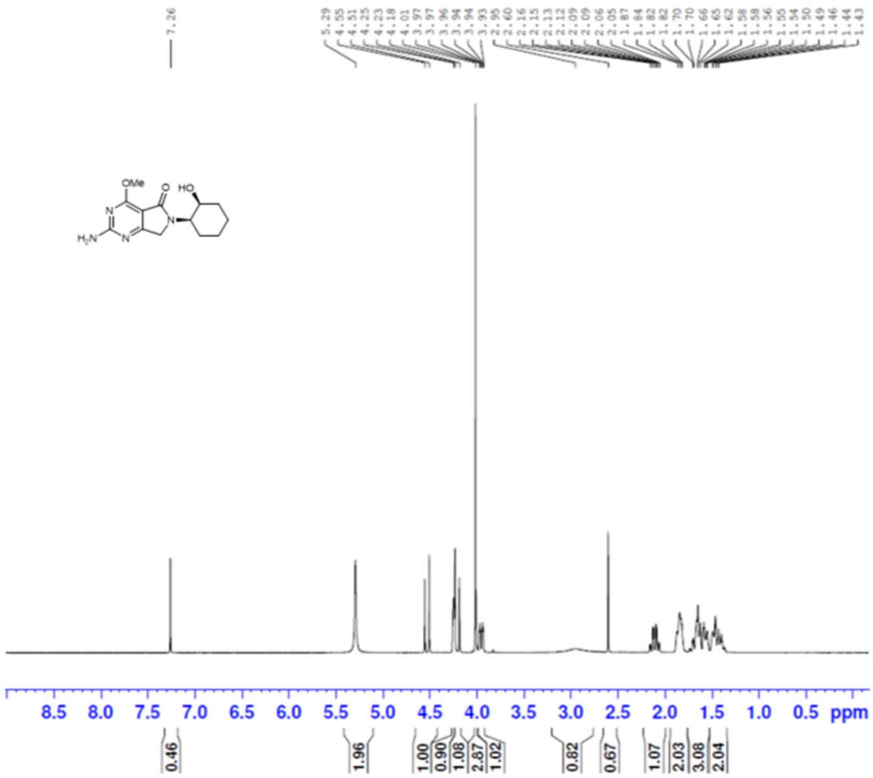

Supplementary Figure 16. ^{1}H NMR spectra of compound **4** (rt, in $CDCl_3$)

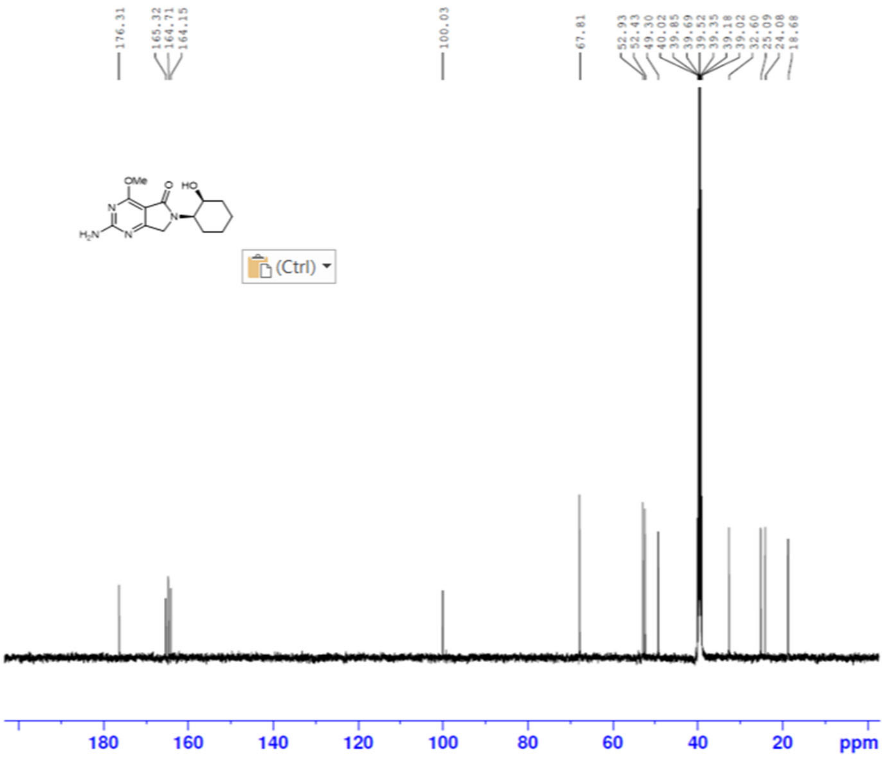

Supplementary Figure 17. ^{13}C NMR spectra of compound **4** (rt, in DMSO-d_6)

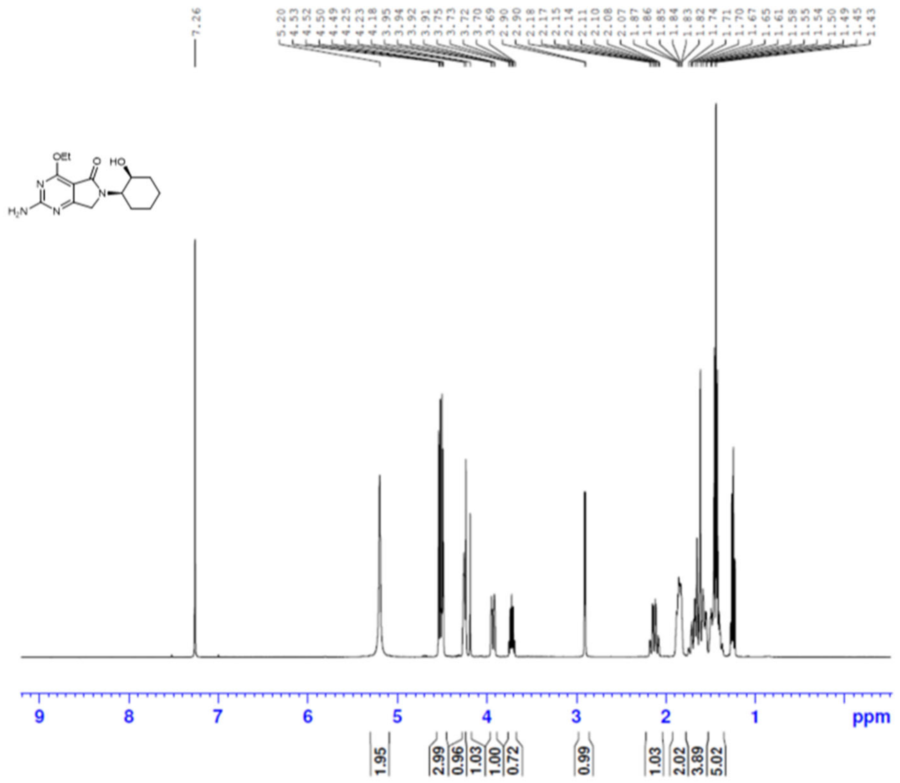

Supplementary Figure 18. ^{1}H NMR spectra of compound **5** (rt, in $CDCl_3$)

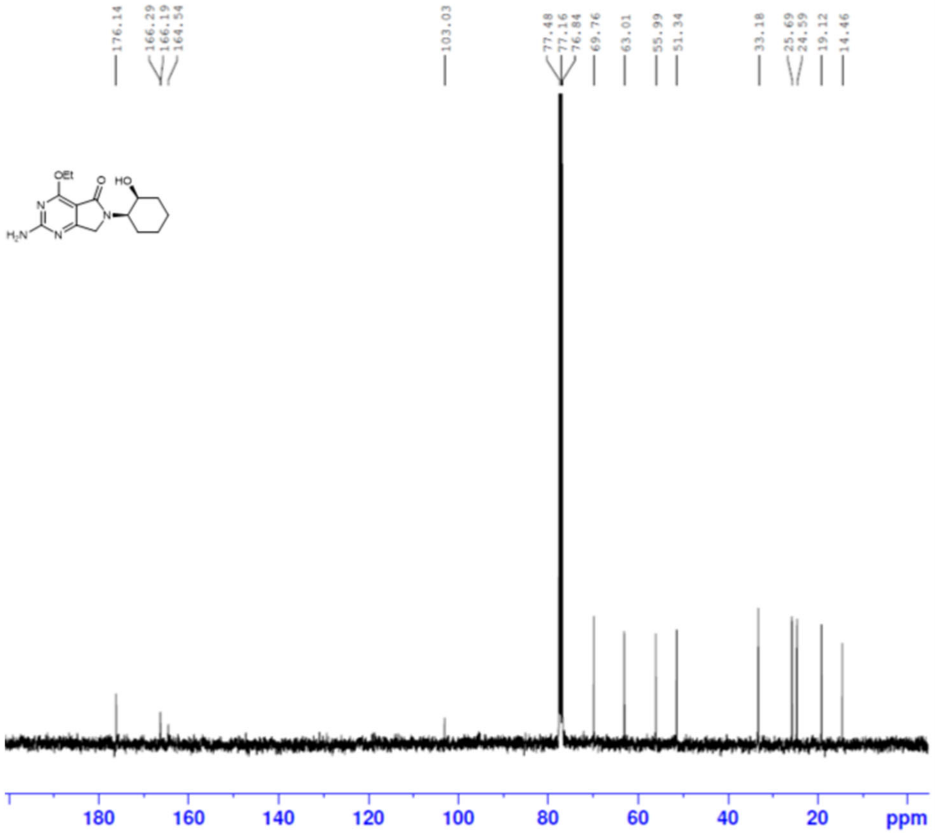

Supplementary Figure 19. ^{13}C NMR spectra of compound **5** (rt, in $CDCl_3$)

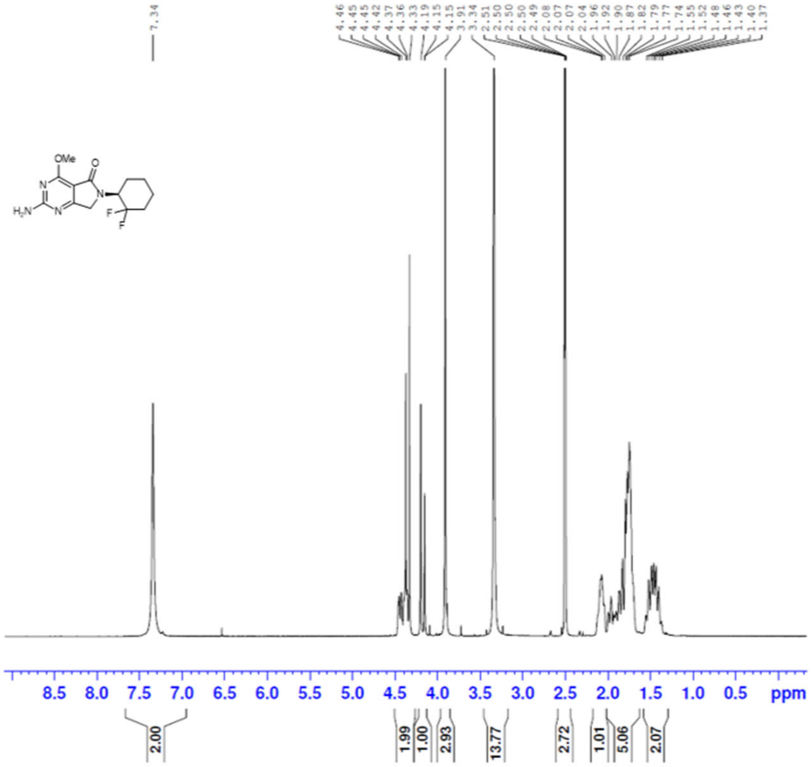

Supplementary Figure 20. ^{1}H NMR spectra of compound **6** (rt, in DMSO-d_6)

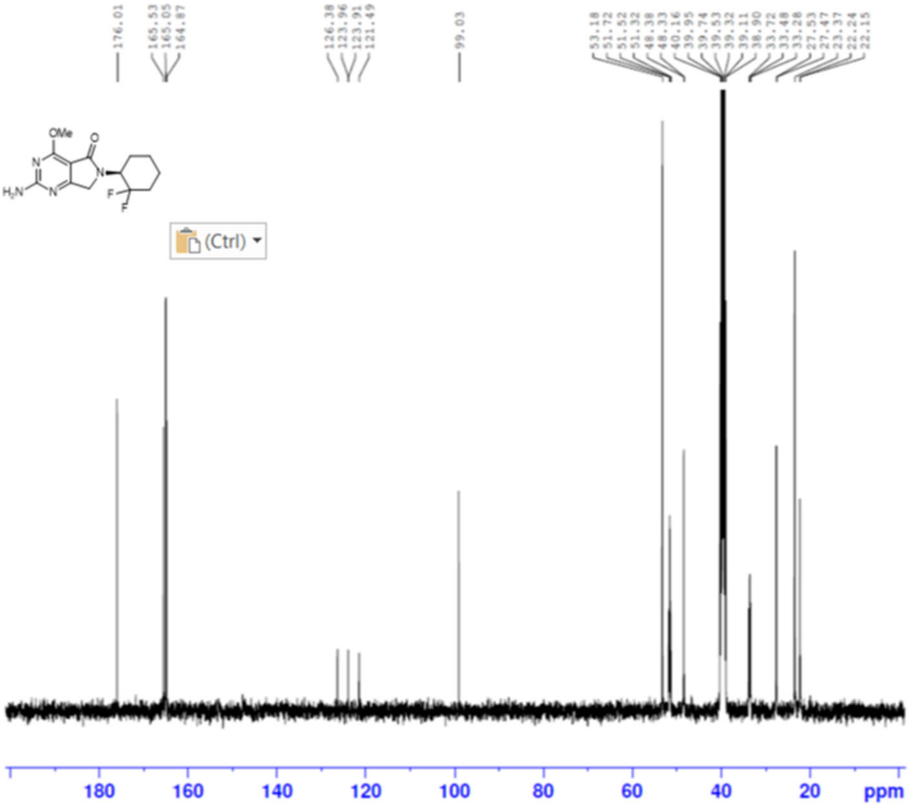

Supplementary Figure 21. ^{13}C NMR spectra of compound **6** (rt, in DMSO-d_6)

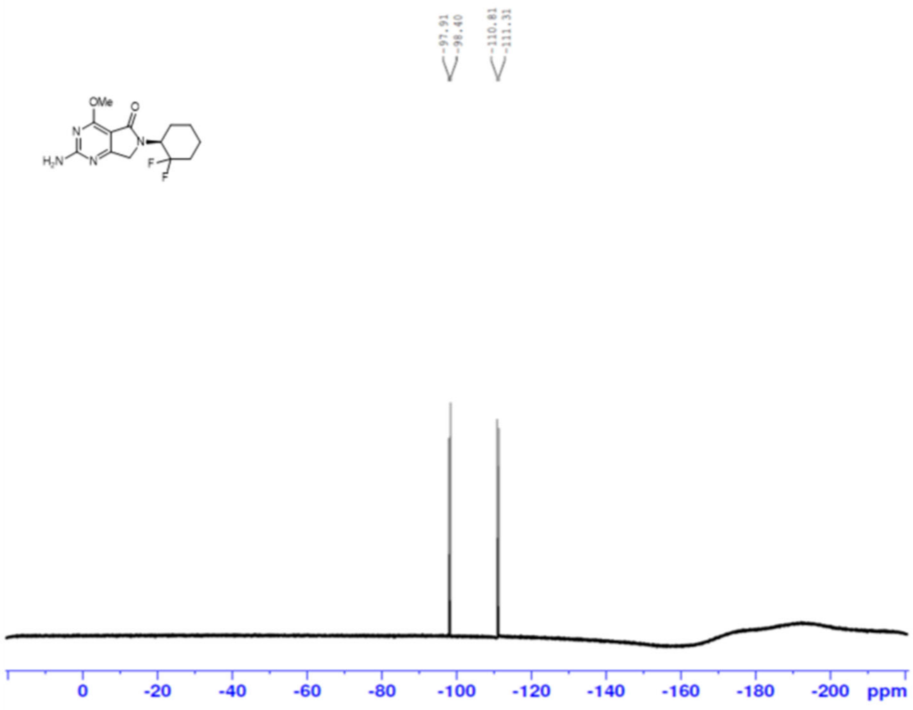

Supplementary Figure 22. ^{19}F NMR spectra of compound **6** (rt, in DMSO-d_6)

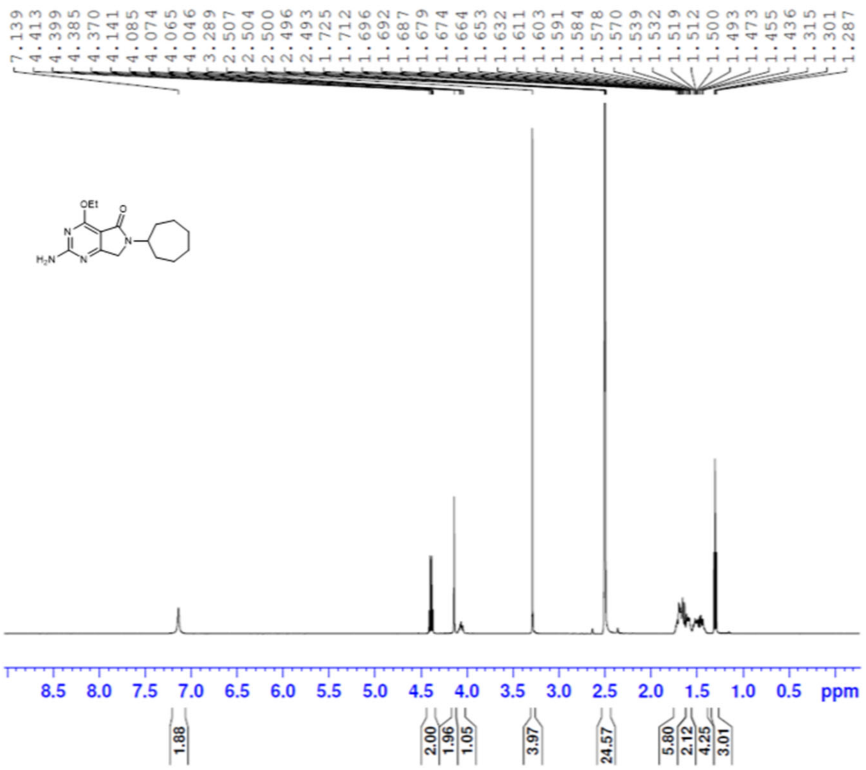

Supplementary Figure 23. ^{1}H NMR spectra of compound **7** (rt, in DMSO-d_6)

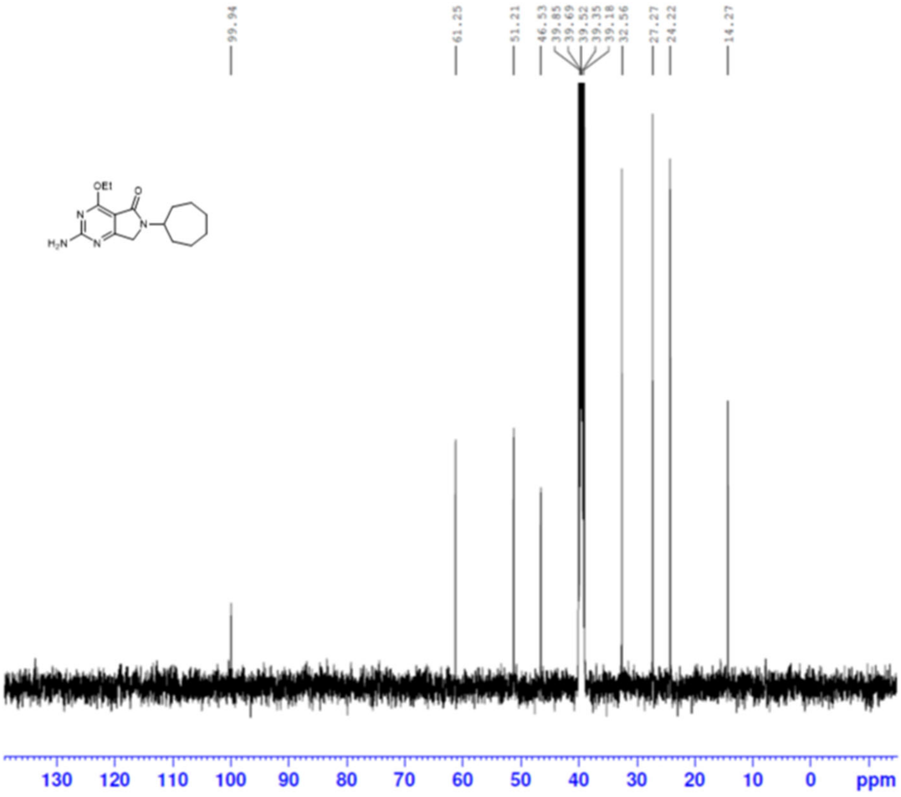

Supplementary Figure 24. ^{13}C NMR spectra of compound **7** (rt, in DMSO-d_6)

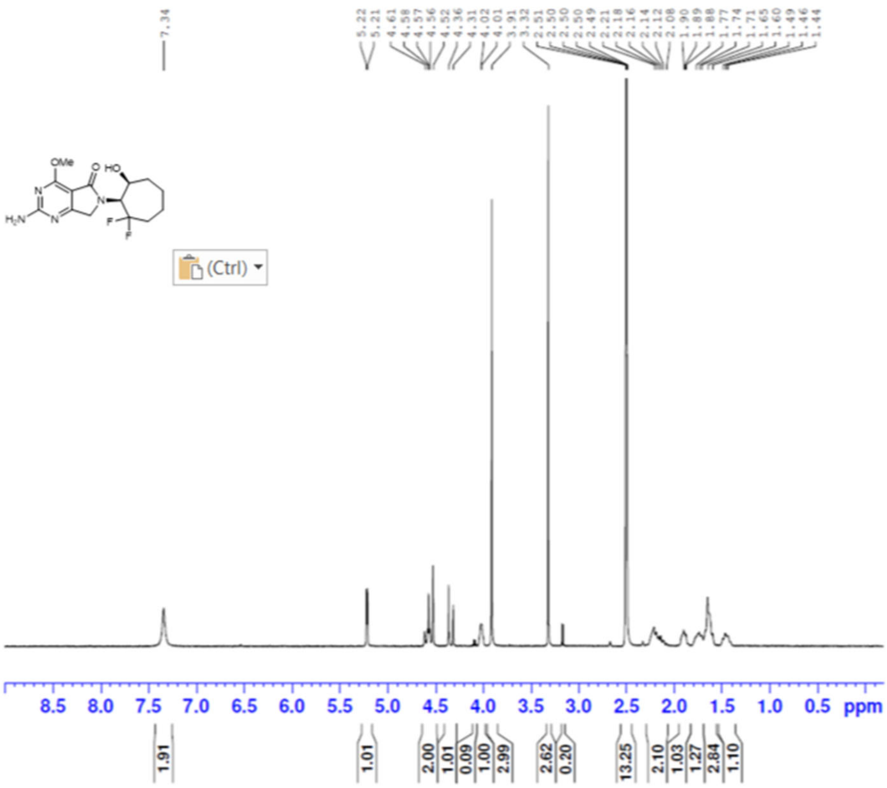

Supplementary Figure 25. ^{1}H NMR spectra of compound **8** (rt, in DMSO-d_6)

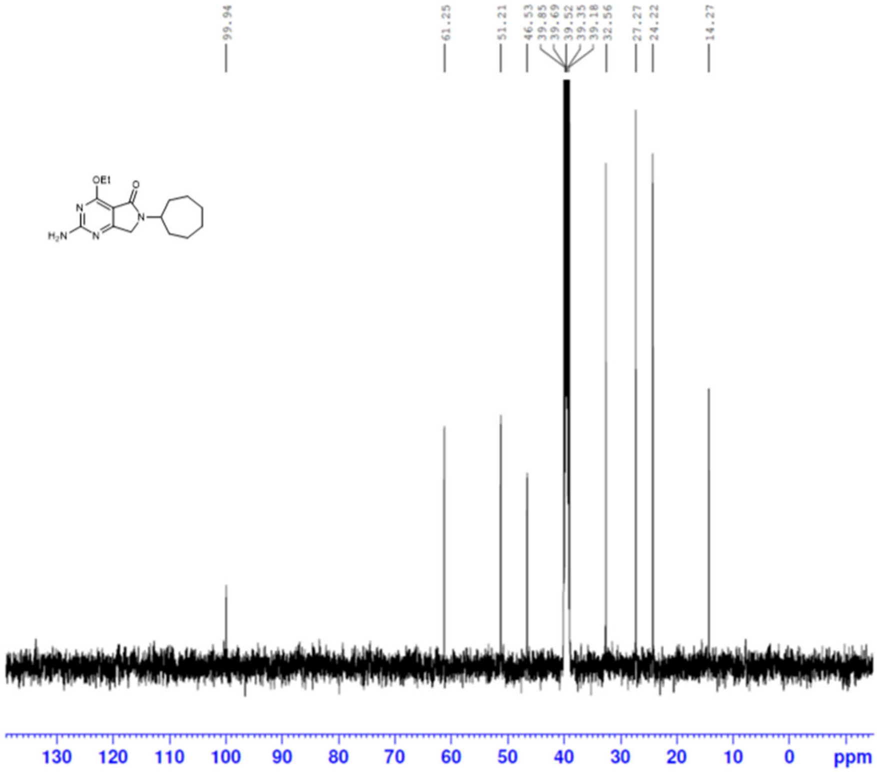

Supplementary Figure 26. ^{13}C NMR spectra of compound **8** (rt, in DMSO-d_6)

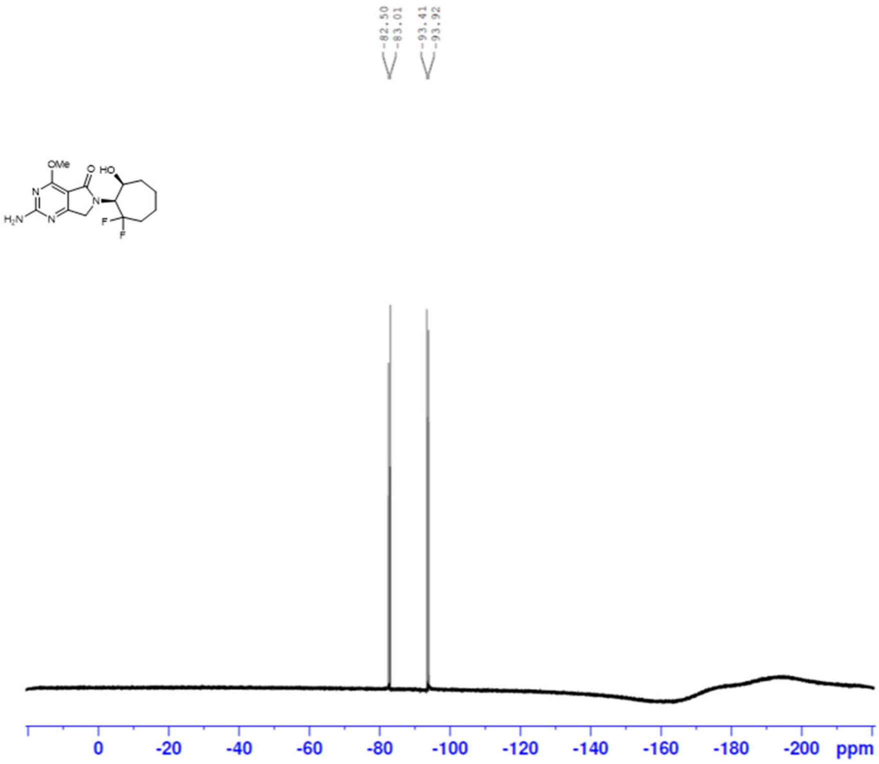

Supplementary Figure 27. ^{19}F NMR spectra of compound **8** (rt, in DMSO-*d*$_6$)

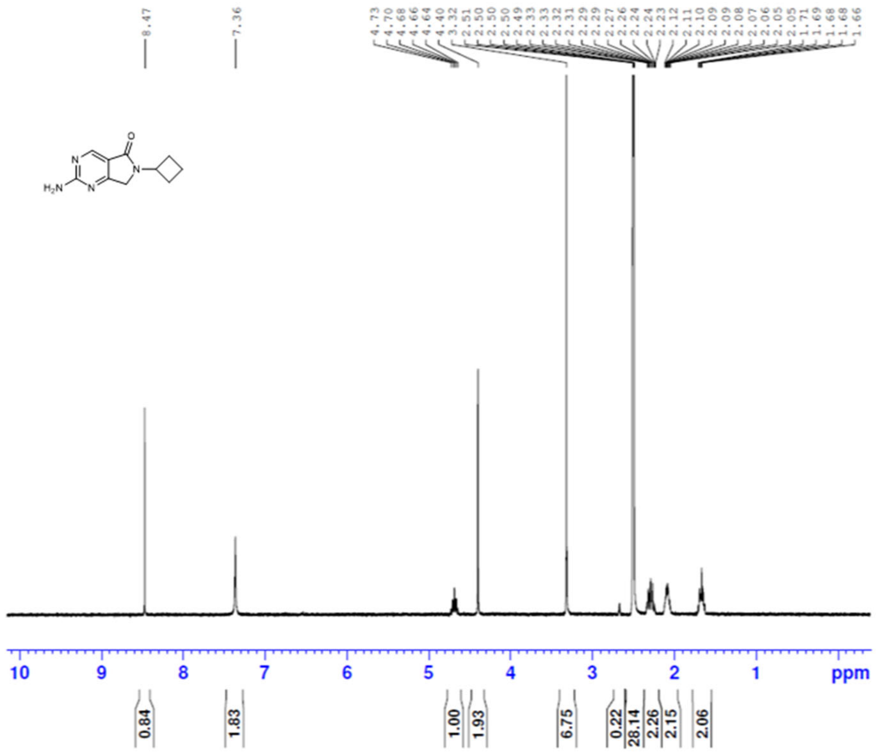

Supplementary Figure 28. ^{1}H NMR spectra of compound **9** (rt, in DMSO-d_6)

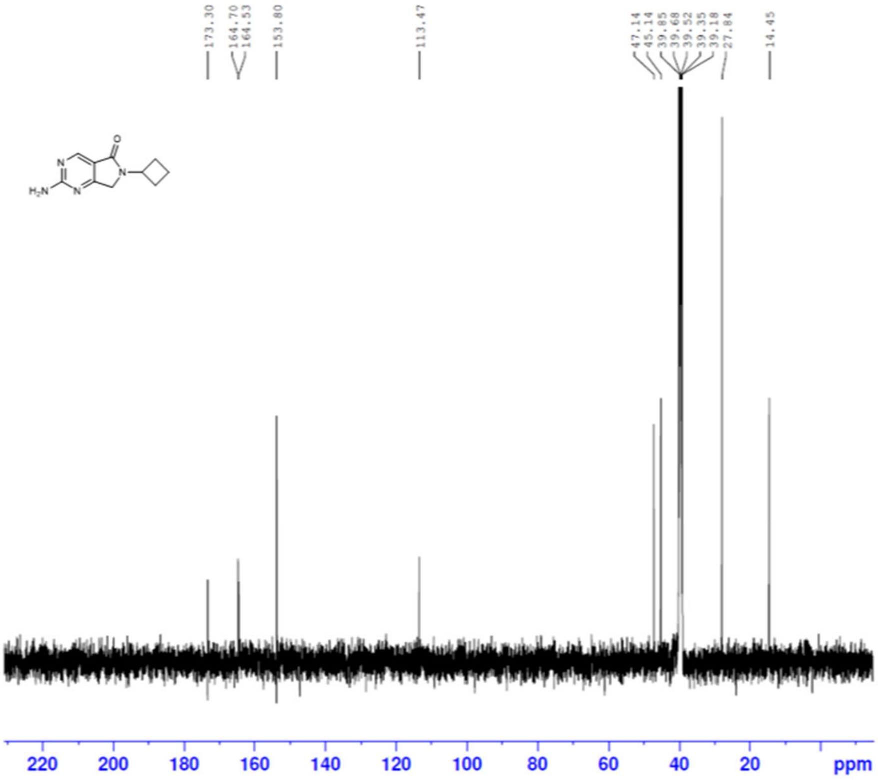

Supplementary Figure 29. ^{13}C NMR spectra of compound **9** (rt, in DMSO-d_6)

Supplementary Figures 30 - 37 HRMS for Compounds

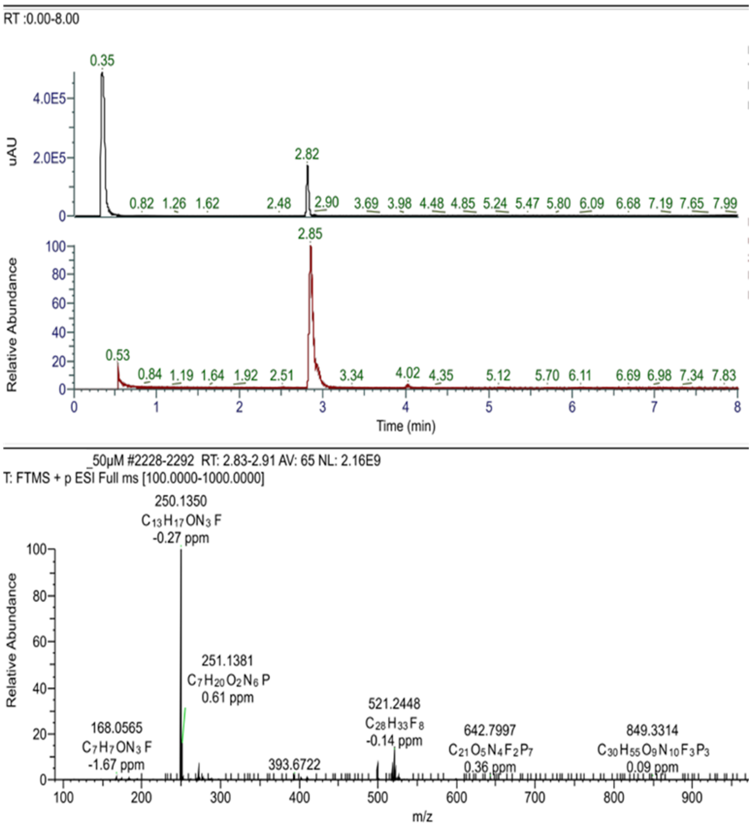

Supplementary Figure 30: HRMS of compound **2**

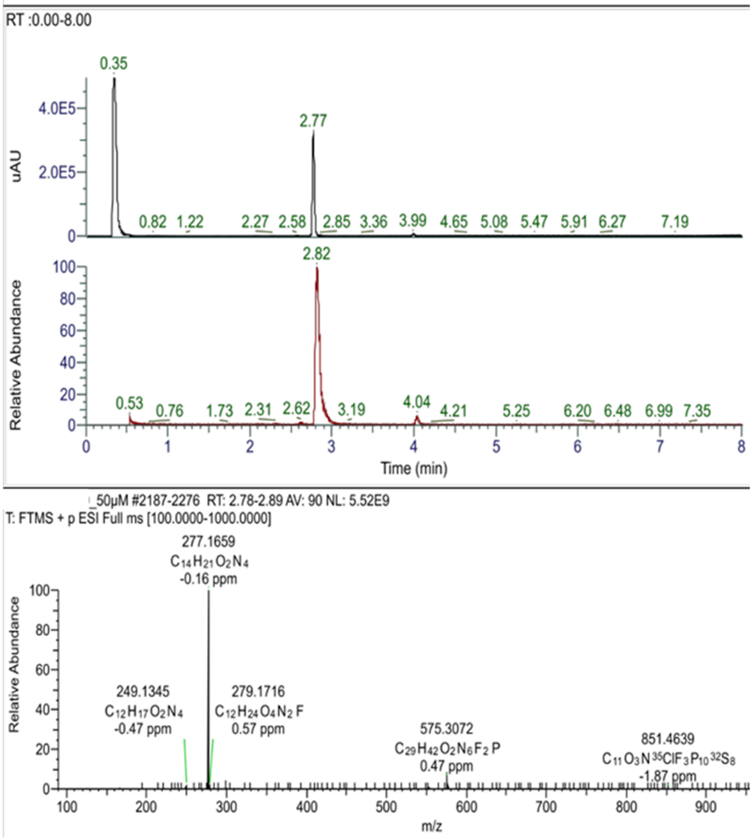

Supplementary Figure 31: HRMS of compound **3**

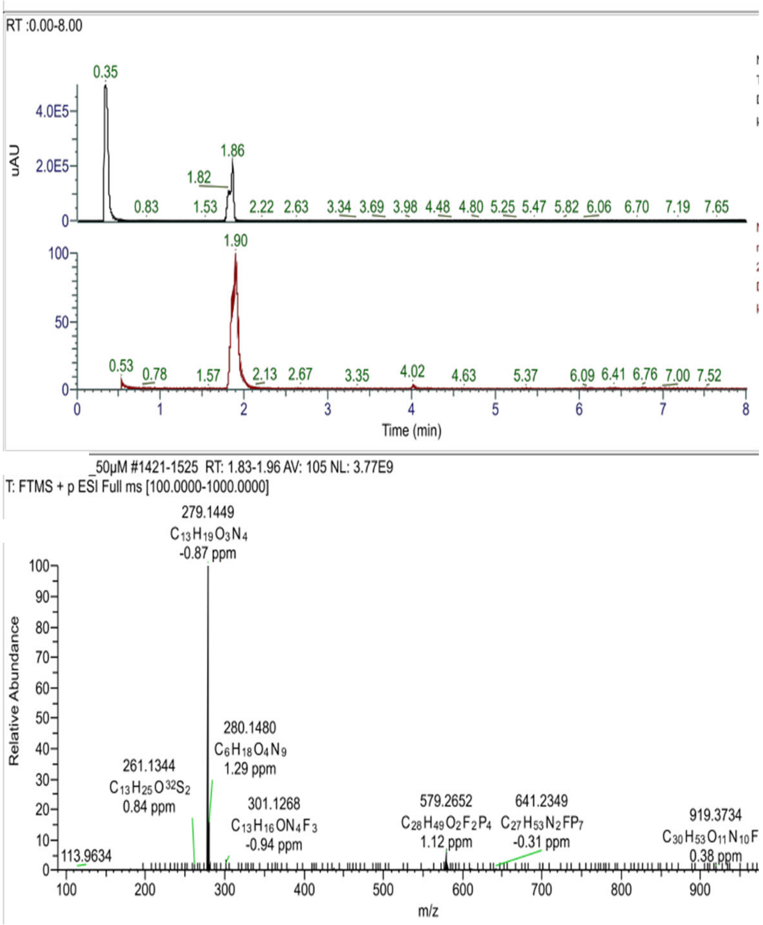

Supplementary Figure 32: HRMS of compound **4**

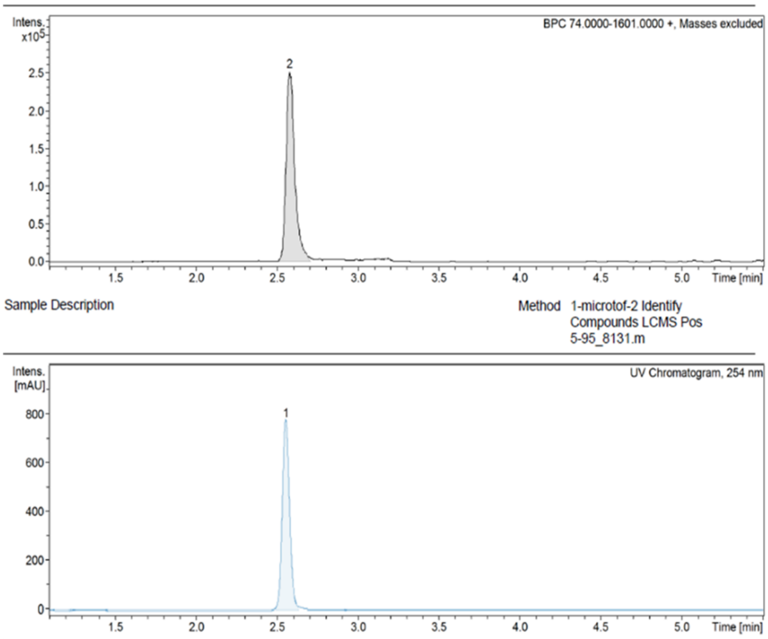

meas. m/z	theo. m/z	|err| [ppm]	mSigma	Formula	Purity(BPC)[%]
293.1618	293.1608	3.2	3	C14 H21 N4 O3	100.0

Supplementary Figure 33: HRMS of compound **5**

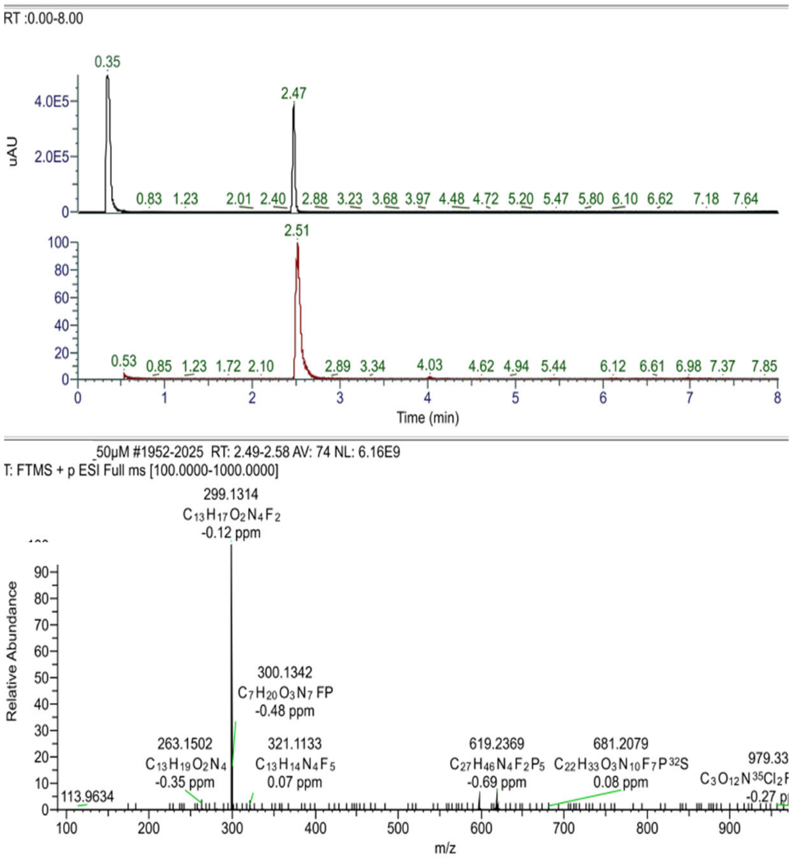

Supplementary Figure 34: HRMS of compound **6**

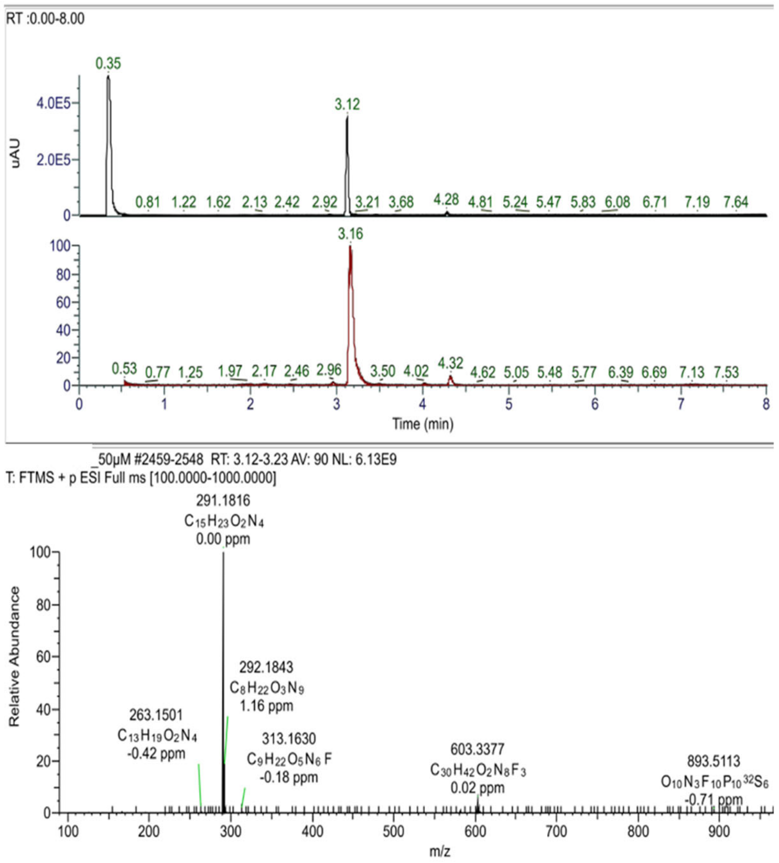

Supplementary Figure 35: HRMS of compound **7**

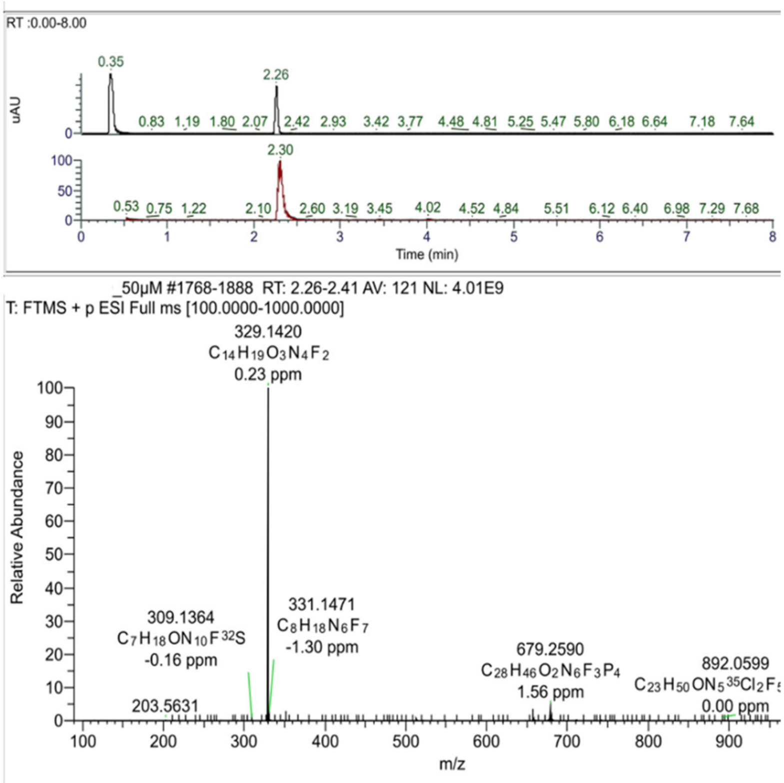

Supplementary Figure 36: HRMS of compound **8**

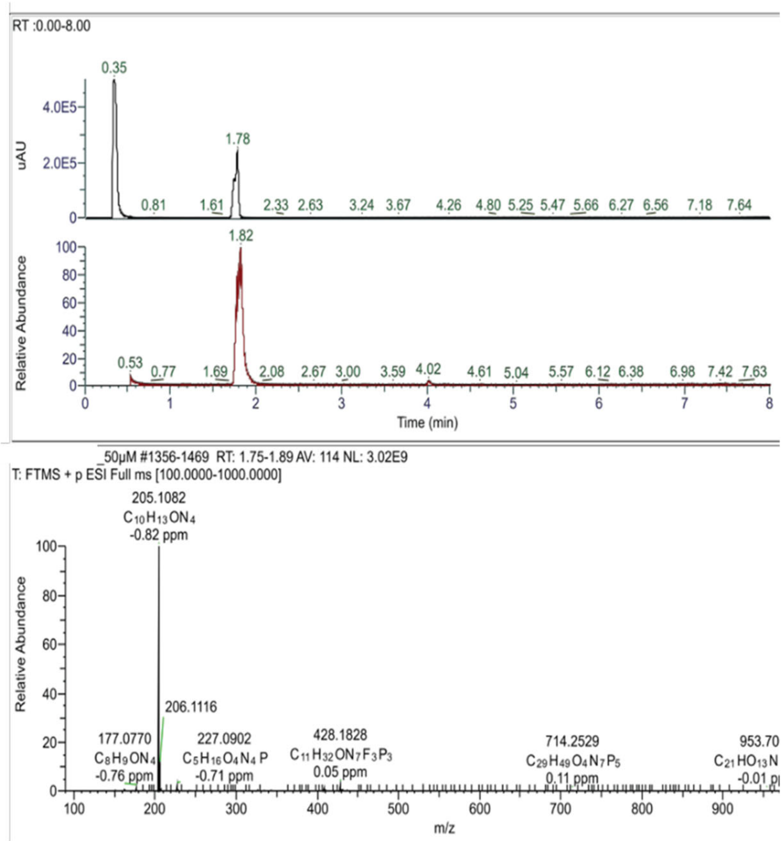

Supplementary Figure 37: HRMS of compound **9**

	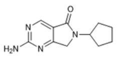	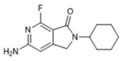	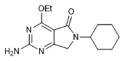	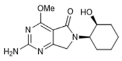	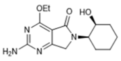	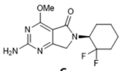	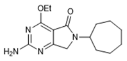	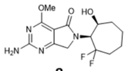
	1	2	3	4	5	6	7	8
Micro clearance (mL/min/g)	<0.5	2.2	1.6	<0.5	<0.5	<0.5	1.4	<0.5
Hepato clearance (mL/min/g)			5.4	<0.5	0.6	<0.6	3.9	<0.7
Cmax (ng/mL)			5306		4261		4796	3699
AUC (µg/mL.min)			223		396		266	499
Clb (mL/min/Kg)			19		23		16	15
Vd (L/Kg)			0.4		0.7		0.4	1
F(%)			43		91		43	74
ED_{99} (mg/kg)					49	134		12

Supplementary Table 1 : ADME properties for series.

In vitro metabolic stability in murine microsomes and hepatocytes for compounds from the series and comparative murine pharmacokinetic parameters.

	Kinetic solubility (µM)	PAMPA-pH7.4 permeability (nm/s)	Plasma protein binding (% bound)				Microsomal clearance (mL/min/g)				Hepatocyte clearance (mL/min/g)			
			Mouse	Rat	Dog	Human	Mouse	Rat	Dog	Human	Mouse	Rat	Dog	Human
**5**	235	24	27	21	28	24	<0.5	<0.5	ND	<0.5	0.6	<0.5	0.8	<0.5
**8**	248	32	35	33	24	44	<0.5	<0.5	<0.5	<0.5	<0.7	<0.5	<0.5	<0.5

Supplementary Table 2: ADME properties for series.

In vitro ADME data including solubility, permeability and cross species metabolic stability and plasma protein binding.

	MIC (µM)	
	No Lysine	+ 1mM Lysine
**5**	1.01	0.87
**8**	0.08	0.08
**Moxifloxacin**	1.23	2.75
**Isoniazid**	3.20	2.72

Supplementary Table 3: Analysis of the impact of lysine on sensitivity to LysRS inhibitors.
MIC was determined for H37Rv ±1mM lysine. The experiment was run once with duplicate samples. Source data are provided as a Source Data file.

Compound	WT H37Rv MIC (µM)	Resistant Mutant MIC (µM)	MIC Ratio (Mutant/WT)	WT LysS IC_{50} (µM)	Resistant Mutant LysS IC_{50} (µM)	IC_{50} Ratio (Mutant/WT)
**2**	0.82	0.1	0.13	13.5	2.39	0.18
**7**	0.89	25	28	0.27	9.90	36
**5**	1.05	35	32	0.82	31.6	39
**8**	0.05	0.18	3.5	0.05	0.25	4.6

Supplementary Table 4: Impact of the resistant mutation on compound sensitivity.
Resistant strain generated against **7** was tested for inhibition of growth by other molecules from the series. The *lysS* mutation from the resistant strain was engineered into an in vitro expression construct and the protein was expressed in E. coli for testing in the standard LysRS in vitro enzyme assay against molecules from the series. Data is from at least two separate experiments.

	Mean IC_{50} (µM)		
**Compound**	**COX1**	**SDHA**	**Cell viability**
**2**	3.6	3.3	3.3
**7**	>100	>100	>100
**5**	>100	>100	>100
**8**	>100	>100	>100
**Linezolid**	16	>100	>100
**Chloramphenicol**	4.6	>100	>100

Supplementary Table 5: LysRS inhibitors do not affect mitochondrial protein synthesis.
Analysis of series representatives in a human mitochondrial protein synthesis assay to highlight potential clinical toxicity that is known to affect other proteins synthesis inhibitors such as linezolid and chloramphenicol. Results are an average of two independent experiments run with duplicate samples.

	MIC (µM)	
**Genus_Species_Strain**	**5**	**8**
Mycobacterium tuberculosis H37Rv	1.25	0.06
Staphylococcus aureus WCUH29	>430	>390
S. aureus PVL-6	>430	>390
Streptococcus pneumoniae ERY2	219	12
S. pneumoniae TPS3	110	24
Streptococcus pyogenes 1308007P	219	24
Haemophilus influenzae H128	430	12
Escherichia coli 7623	>430	>390
E. coli NCTC 13441	>430	>390
Klebsiella pneumoniae 1161486	>430	>390
K. pneumoniae VA-361	>430	>390
Pseudomonas aeruginosa PAO1 (MV)	>430	>390
P. aeruginosa SR27001	>430	>390
Acinetobacter baumannii BM4454	>430	>390
A. baumannii 1485247	>430	>390

Supplementary Table 6: Antibacterial activity for 5 & 8.

Comparative antibacterial activity for **5** & **8** against *M. tuberculosis* and other clinically relevant Gram positive and negative bacterial strains. Profiling against this panel of strains was performed in singlicate.

	**5**	**8**	**BDQ**	**PRE**	**RIF**	**INH**
**H37Rv**	0.04	0.60	0.30	0.07	0.01	0.39
**H37Rv (atpE:E61D)**	0.02	0.30	3.13	0.04	0.05	0.39
**Clinical strain (Rv0678:G25E)**	0.02	0.39	1.20	0.04	0.05	0.30
**Clinical strain (Rv0678:A153D)**	0.02	0.39	1.20	0.04	0.05	0.30
**H37Rv (ddn:P86N)**	0.02	0.39	0.07	37	0.05	0.39
**H37Rv (fgd:R187G)**	0.02	0.39	0.07	>50	0.05	0.30
**H37Rv (fbiA:10bp del)**	0.02	0.20	0.05	>50	0.05	0.39
**Clinical strain (fbiC:A639P)**	0.07	0.60	0.39	>50	0.30	0.39

Supplementary Table 7: Activity of 5 & 8 against laboratory strains resistant to bedaquiline or pretomanid.

Sensitivity of laboratory strains resistant to Bedaquiline (BDQ) or Pretomanid (PRE) to LysS inhibitors (**5** & **8**), all strains were evaluated in duplicate.

	RESISTANCE PROFILE													MIC (μM)	
**Strain**	**Rifampicin**	**Isoniazid**	**Ethambutol**	**Streptomicin**	**pyrazinamide**	**capreomycin**	**kanamycin**	**Amikacin**	**Moxifloxacin**	**Ciprofloxacin**	**Ofloxacin**	**Ethionamide**	**PAS**	**5**	**8**
WT clinical	S	S	S	S	S	S	S	S	S	S	S	S	S	1.25	0.12
1	**R**	**R**	**R**	S	S	S	S				**R**	S	**R**	0.6	0.03
2	**R**	**R**	**R**	S	S	**R**	**R**				**R**	**R**		0.6	0.03
3	**R**	**R**	**R**	**R**		S	S				**R**	**R**	**R**	0.6	0.03
4	S	**R**	S	**R**	S	S	S	S	S	S	S	S	**R**	0.6	0.03
5	S	S	S	**R**	S	**R**	S	S	**R**	**R**	**R**	S	S	1.25	0.06
6	**R**	**R**	S	S	**R**	S	S	S	**R**	**R**	**R**	S		2.5	0.06
7	S	S	S	S	S	S	S	S	**R**	**R**	**R**	S	S	1.25	0.06
8	**R**	**R**	**R**	**R**	**R**	**R**	**R**			**R**		**R**		1.25	0.06
9	**R**	**R**	**R**	**R**	**R**	**R**	**R**			**R**		**R**		1.25	0.06
10	**R**	S	S	S	**R**	**R**	**R**			**R**		S		1.25	0.06
11	S	S	S	S	S	S	**R**			**R**	S	S		1.25	0.06
12	S	S	S	S	S	S	S			**R**		S		1.25	0.06
13	**R**	**R**	**R**	**R**	**R**	S	S			**R**		**R**		1.25	0.03
14	**R**	**R**	**R**	**R**	**R**	**R**	**R**			**R**		**R**		0.3	0.02
15	**R**	**R**	**R**	**R**	**R**	**R**	**R**			**R**		**R**		1.25	0.03
16	**R**	**R**	**R**	**R**	**R**	**R**	**R**		**R**		**R**	S		1.25	0.06
17	**R**	**R**	**R**	S	**R**	S	S			**R**		**R**		1.25	0.06
18	**R**	S	S	**R**	S	S	S			**R**		S		0.6	0.06
19	**R**	**R**	**R**	**R**	**R**	S	S		**R**		**R**	**R**		0.6	0.03
20	**R**	**R**	**R**	**R**	**R**	S	S			**R**		S		0.3	0.02
21	S	**R**	S	S	**R**	S		S	**R**		**R**	S		1.25	0.06
22	**R**	**R**	**R**	**R**	**R**	**R**		**R**	S		S	**R**		0.6	0.02
23	**R**	**R**	**R**	**R**	**R**	**R**	**R**			**R**		S	S	1.25	0.06
24	S	S	S	S	S	S	S			**R**	**R**	S	S	1.25	0.03
25	**R**	**R**	**R**	**R**	S	S	**R**			**R**		**R**		1.25	0.06
26	**R**	**R**	**R**	**R**	**R**	S		S	**R**		**R**	S		1.25	0.06
27	S	**R**	**R**	S	**R**	S	S			S	**R**	S	**R**	1.25	0.03
28	**R**	**R**	**R**	**R**	S	**R**	**R**		S	**R**	**R**	S	S	1.25	0.06
29	**R**	**R**	**R**	**R**		**R**	**R**				**R**	**R**		1.25	0.06
30	**R**	**R**	**R**	**R**	**R**	S	S	S	**R**		**R**	**R**		0.3	0.02
H37Rv	S	S	S	S	S	S	S	S	S	S	S	S	S	1.25	0.06

Supplementary Table 8: Activity of 5 & 8 against clinical samples.
Sensitivity of WT and drug resistant clinical strains of *M. tuberculosis* to **5** & **8.** All strains were from the Vall d'hebron Hospital clinical isolate collection and are marked as either sensitive (s) or resistant (**R**) to selected drugs. Profiling against this panel of strains was performed in singlicate.

	Lysine only	**2**	**8**
**Data collection**			
Space group	$P4_12_12$	$P4_12_12$	$P4_12_12$
Cell dimensions			
a,*b*, *c* (Å)	83.84, 83.84, 147.78	82.89, 82.89, 146.62	84,20, 84.20, 147.98
α, β, γ (°)	90, 90, 90	90, 90, 90	90, 90, 90
Resolution (Å)	73.89-1.92 (1.95-1.92)	82.89-2.58 (2.62-2.58)	49.33-2.20 (2.27-2.20)
R_{merge}	0.099 (3.452)	0.314 (3.215)	0.205 (1.722)
$I / \sigma I$	15.1 (1.0)	4.6 (0.9)	5.8 (1.1)
CC1/2	1.0 (0.7)	1.0 (0.7)	0.995 (0.857)
Completeness (%)	100 (99.6)	100 (99.1)	100 (100)
Redundancy	13.8 (12.0)	8.3 (8.8)	11.1 (10.5)
**Refinement**			
Resolution (Å)	1.92	2.58	2.20
No. reflections	38856	15842	26375
R_{work} / R_{free}	21.09/24.79	29.29 / 33.91	24.38 / 30.47
No. atoms			
Protein	3547	3504	3591
Ligand / lysine	6 (glycerol) / 10	18/10	23 / 10
Water	131	72	69
B-factors			
Protein	52.85	64.20	64.6
Ligands / Lysine	38.4 / 52.6	48.9 / 52.8	74.3 / 55.6
Water	44.26	34.08	46.9
R.m.s. deviations			
Bond lengths (Å)	0.0082	0.0047	0.0054
Bond angles (°)	1.5426	1.4757	1.4812
PDB codes	7QH8	7QHN	7QI8

*Values in parentheses are for highest-resolution shell.

Supplementary Table 9: Crystal data collection and refinement statistics.

Supplementary References

1 Hiroshi, B., Toshio, T. & Satoshi, S. Fused heterocyclic ring derivative and use thereof. WO/2011/024871 (2011).

2 Burger, M. *et al.* PIM kinase inhibitors and methods of their use. WO/2008/106692 (2008).

3 Sundén, H. *et al.* Chiral Dihydrobenzofuran Acids Show Potent Retinoid X Receptor–Nuclear Receptor Related 1 Protein Dimer Activation. *J. Med. Chem.* **59**, 1232-1238, doi:10.1021/acs.jmedchem.5b01702 (2016).
