# Supplementary material for: Lysyl-tRNA synthetase, a target for urgently needed M. tuberculosis drugs
Source: Nat Commun. 2022 Oct 11;13:5992. doi: 10.1038/s41467-022-33736-5 (PMC9552147; doi:10.1038/s41467-022-33736-5)
Supplement: Supplementary file 3 — Reporting summary [file 41467_2022_33736_MOESM3_ESM.pdf]

nature portfolio

Corresponding author(s): Cleghorn LAT

Last updated by author(s): Sep 15, 2022

Reporting Summary

Nature Portfolio wishes to improve the reproducibility of the work that we publish. This form provides structure for consistency and transparency in reporting. For further information on Nature Portfolio policies, see our Editorial Policies and the Editorial Policy Checklist.

Statistics

For all statistical analyses, confirm that the following items are present in the figure legend, table legend, main text, or Methods section.

n/a	Confirmed	
☐	☒	The exact sample size (*n*) for each experimental group/condition, given as a discrete number and unit of measurement
☐	☒	A statement on whether measurements were taken from distinct samples or whether the same sample was measured repeatedly
☐	☒	The statistical test(s) used AND whether they are one- or two-sided<br>*Only common tests should be described solely by name; describe more complex techniques in the Methods section.*
☒	☐	A description of all covariates tested
☒	☐	A description of any assumptions or corrections, such as tests of normality and adjustment for multiple comparisons
☐	☒	A full description of the statistical parameters including central tendency (e.g. means) or other basic estimates (e.g. regression coefficient) AND variation (e.g. standard deviation) or associated estimates of uncertainty (e.g. confidence intervals)
☐	☒	For null hypothesis testing, the test statistic (e.g. *F*, *t*, *r*) with confidence intervals, effect sizes, degrees of freedom and *P* value noted<br>*Give P values as exact values whenever suitable.*
☒	☐	For Bayesian analysis, information on the choice of priors and Markov chain Monte Carlo settings
☒	☐	For hierarchical and complex designs, identification of the appropriate level for tests and full reporting of outcomes
☒	☐	Estimates of effect sizes (e.g. Cohen's *d*, Pearson's *r*), indicating how they were calculated

Our web collection on statistics for biologists contains articles on many of the points above.

Software and code

Policy information about availability of computer code

Data collection	Crystal data were collected using GDA (Diamond) and MXCUBE (Soleil).
Data analysis	Compound potencies against LysRS, KARS1, HepG2 cytotoxicity, MIC and IntraMac IC90 were calculated in IDBS Activitybase as were in vitro clearance values. Mycobacterium tuberculosis resistant strain growth and in vivo efficacy data was analysed using Graphpad prism (V 6 and 9). DNA sequence alignments were performed with bwa mem (0.7.17-r1188), samtools (1.11)and Picard tools (2.25.4). The metabolomic experiment was analyzed using Agilent ProFinder and Mass Hunter Qual and the image was created using TreeView. Mouse and Rat PK analysis was performed with PK Solutions while the dog used Win Nonlin. X DS and DIALS were used for crystal data integration. Various software incorporated into the CCP4 (7.1) suite as used for analysis and structure refinement: AIMLESS was used for scaling and merging; Phaser was used for molecular replacement; REFMAC5 was used for structure refinement, and AceDRG was used for ligand geometry. Coot was used for model building and refinement. PyMOL 2.5.2 was used to make crystallographic figures.

For manuscripts utilizing custom algorithms or software that are central to the research but not yet described in published literature, software must be made available to editors and reviewers. We strongly encourage code deposition in a community repository (e.g. GitHub). See the Nature Portfolio guidelines for submitting code & software for further information.

Data

Policy information about availability of data

All manuscripts must include a data availability statement. This statement should provide the following information, where applicable:

- Accession codes, unique identifiers, or web links for publicly available datasets
- A description of any restrictions on data availability
- For clinical datasets or third party data, please ensure that the statement adheres to our policy

The M. tuberculosis str. H37RvCO; NCBI Reference Sequence: NZ_CM001515.1 was used for sequence alignments. The three crystal structures have been deposited in the protein data bank under the codes 7QH8, 7QHN and 7QI8. For cross species comparison of crystal structures with other lysyl-tRNA synthetases the following PDB crystal structures were used 3bju, 6ild, 6agt & 1e1t. The authors declare that data supporting the findings of this study are available within the paper and its supplementary information files. Source data are provided with this paper.

Field-specific reporting

Please select the one below that is the best fit for your research. If you are not sure, read the appropriate sections before making your selection.

☒ Life sciences ☐ Behavioural & social sciences ☐ Ecological, evolutionary & environmental sciences

For a reference copy of the document with all sections, see nature.com/documents/nr-reporting-summary-flat.pdf

Life sciences study design

All studies must disclose on these points even when the disclosure is negative.

Sample size	Sample sizes are given in the manuscript. Based on our historical experiments the numbers of mice used in the acute and chronic defficacy studies have been shown to have enough power to detect significant differences.
Data exclusions	No data was excluded.
Replication	Replication numbers are reported in the text or in the methodology sections. All in vitro experiments were performed at least in duplicate. Where appropriate a measure of the error is reported. All attempts at replication were successful.
Randomization	Infected mice for the efficacy studies were randomly allocated into experimental groups. In vitro experiments were not randomized as samples were not grouped
Blinding	For the mouse efficacy studies, the same investigators carried out the animal infection, dosing of test compound and analysis so were not blinded to group allocation. For in vitro experiments blinding was not performed as the in vitro readouts do not require researcher based judgements

Reporting for specific materials, systems and methods

We require information from authors about some types of materials, experimental systems and methods used in many studies. Here, indicate whether each material, system or method listed is relevant to your study. If you are not sure if a list item applies to your research, read the appropriate section before selecting a response.

Materials & experimental systems

n/a	Involved in the study	
☒	☐	Antibodies
☐	☒	Eukaryotic cell lines
☒	☐	Palaeontology and archaeology
☐	☒	Animals and other organisms
☒	☐	Human research participants
☒	☐	Clinical data
☒	☐	Dual use research of concern

Methods

n/a	Involved in the study	
☒	☐	ChIP-seq
☒	☐	Flow cytometry
☒	☐	MRI-based neuroimaging

Eukaryotic cell lines

Policy information about cell lines

Cell line source(s)	HepG2 & THP-1 cells were obtained directly from the European Collection of Authenticated Cell Cultures (ECACC) and the American Type Culture Collection

Authentication	ECACC/ATCC certifies authenticity of their cell lines. No internal authentification was performed
Mycoplasma contamination	Cells were mycoplasma tested and confirmed negative.
Commonly misidentified lines (See ICLAC register)	None

Animals and other organisms

Policy information about studies involving animals; ARRIVE guidelines recommended for reporting animal research

Laboratory animals	Female C57BL/6 mice (8-10 week-old), Female BALB/c mice (4-6 weeks old), Male Hans Wistar rats (6-8 weeks old) & Male beagle dogs (6 months-4 years old)
Wild animals	The study did not involve wild animals
Field-collected samples	The study did not involve field samples
Ethics oversight	University of Dundee Ethical Review Committee, WuXi AppTec Co., Ltd Animal Care Committee, GSK Institutional Animal Care and Use Committee, Johns Hopkins University Animal Care and Use Committee

Note that full information on the approval of the study protocol must also be provided in the manuscript.
